# A Phylogenetic Approach to Structural Variation in Organization of Nuclear Group I Introns and Their Ribozymes

**DOI:** 10.3390/ncrna7030043

**Published:** 2021-07-22

**Authors:** Betty M. N. Furulund, Bård O. Karlsen, Igor Babiak, Steinar D. Johansen

**Affiliations:** 1 Genomic Division, Faculty of Biosciences and Aquaculture, Nord University, 8049 Bodø, Norway; betty.m.furulund@nord.no (B.M.N.F.); igor.s.babiak@nord.no (I.B.); 2 Research Laboratory, Department of Microbiology, Nordland Hospital Trust, 8005 Bodø, Norway; bard.ove.karlsen@nordlandssykehuset.no

**Keywords:** catalytic introns, homing endonuclease, intron biology, intron evolution, intron mobility, myxomycete, ribosomal DNA, ribozyme

## Abstract

Nuclear group I introns are restricted to the ribosomal DNA locus where they interrupt genes for small subunit and large subunit ribosomal RNAs at conserved sites in some eukaryotic microorganisms. Here, the myxomycete protists are a frequent source of nuclear group I introns due to their unique life strategy and a billion years of separate evolution. The ribosomal DNA of the myxomycete *Mucilago crustacea* was investigated and found to contain seven group I introns, including a direct repeat-containing intron at insertion site S1389 in the small subunit ribosomal RNA gene. We collected, analyzed, and compared 72 S1389 group IC1 introns representing diverse myxomycete taxa. The consensus secondary structure revealed a conserved ribozyme core, but with surprising sequence variations in the guanosine binding site in segment P7. Some S1389 introns harbored large extension sequences in the peripheral region of segment P9 containing direct repeat arrays. These repeats contained up to 52 copies of a putative internal guide sequence motif. Other S1389 introns harbored homing endonuclease genes in segment P1 encoding His-Cys proteins. Homing endonuclease genes were further interrupted by small spliceosomal introns that have to be removed in order to generate the open reading frames. Phylogenetic analyses of S1389 intron and host gene indicated both vertical and horizontal intron transfer during evolution, and revealed sporadic appearances of direct repeats, homing endonuclease genes, and guanosine binding site variants among the myxomycete taxa.

## 1. Introduction

Group I introns are intervening sequences that interrupt protein-coding or structural RNA genes in diverse taxa [1]. They appear widespread but sporadic in nature [2], and nuclear group I introns have so far only been found within ribosomal RNA (rRNA) genes at phylogenetically conserved sites in protist or fungal eukaryotic microorganisms [3]. A hallmark of group I introns is the characteristic RNA structure that constitutes the group I ribozyme core responsible for intron removal from the precursor RNA. Here, a series of paired segments, named P1 to P13, are organized into higher order helical stack domains (substrate, scaffold, and catalytic domains) [3,4,5]. Critical structure elements in catalysis, among others, are the U:G wobble pair at the 5′ splice site (SS) in segment P1, and the guanosine binding site (G-site) in segment P7. Two subgroups dominate nuclear group I introns, the group IC1 and group IE introns [3,6,7]. These subgroups are easily distinguished due to notable differences in the scaffold domain and the G-site. Crystal structures and three-dimensional models of the *Tetrahymena thermophila* group IC1 ribozyme and the *Didymium iridis* group IE ribozyme, respectively, have been published [8,9].

Group I intron self-splicing is catalyzed by the group I ribozyme and follows a two-step reaction pathway initiated by a nucleophilic attack of exogenous guanosine (exoG) bound to the G-site [reviewed in 1]. Here, exoG attacks the 5′ SS and becomes covalently ligated to the 5′ end of intron RNA. In the second reaction the terminal intron nucleotide (ωG) replaces exoG at the G-site and becomes attacked by the free 3′ hydroxyl group of the upstream exon. The outcome of splicing is exon ligation and intron excision. In addition, some nuclear group I introns generate circular intron RNA molecules, either as truncated circles [10] or full-length circles [11].

Homing at the DNA-level is an efficient intron mobility process linked to sexual mating. Group I intron homing is initiated by a double-strand break at the intron-lacking allele generated by an intron-encoded homing endonuclease and completed by gene conversion using the intron-containing allele as a template [reviewed in 3]. There are several distinct families of homing endonucleases, and mobile nuclear group I introns encode homing endonucleases of the His-Cys family [12,13]. Homing by nuclear group I introns has only been shown experimentally in the myxomycetes *Physarum polycephalum* and *D. iridis* [14,15]. Reverse splicing at the RNA-level may also contribute to intron mobility into entopic (homing) or ectopic (transposition) sites [3,16]. Reverse splicing has been reported in some nuclear group I introns [17,18], but experimental support of genomic integration is still lacking.

Myxomycetes (plasmodial slime molds) are unicellular eukaryotes classified as a distinct and ancient protist phylum [19]. The myxomycete ribosomal DNA (rDNA) minichromosome is a frequent host of nuclear group I introns, and 23 genic sites in the small subunit (SSU) and large subunit (LSU) rRNA genes are known to harbor intron insertions [3,20]. *Mucilago* is a genus in the order Physarales that contains only one species, *M. crustacea* [21]. We have isolated and characterized the nuclear rRNA genes in a specimen of *M. crustacea* collected in sub-Arctic Norway. DNA sequencing revealed seven group I introns; two in the SSU rRNA gene (insertion sites S788 and S1389) and five in the LSU rRNA gene (insertion sites L828, L1926, L1949, L2066, and L2449). The intron at insertion site S1389 contained a complex structural organization and was studied in more detail. In an earlier report we showed that the myxomycete S1389 introns constituted a monophyletic clade (the S1389 family) among nuclear group I introns [22]. Here we extended our analyses to involve 72 myxomycete S1389 group I introns identified in a variety of myxomycete taxa representing four taxonomic orders.

## 2. Results

### 2.1. The Nuclear rDNA of Mucilago Crustacea Harbors Multiple Group I Intron Insertions

**Figure 1:**
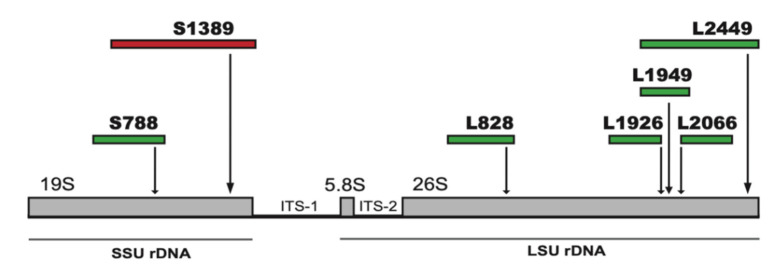
Schematic representation of nuclear rDNA of *Mucilago crustacea.* The SSU rDNA (19S rRNA gene region) is interrupted by group I introns at positions S788 and S1389. The LSU rDNA (5.8S + 26S rRNA gene regions) is interrupted by five group I introns at positions L828, L1926, L1949, L2066, and L2449. See GenBank accession number MZ313554 for details.

**Figure 2:**
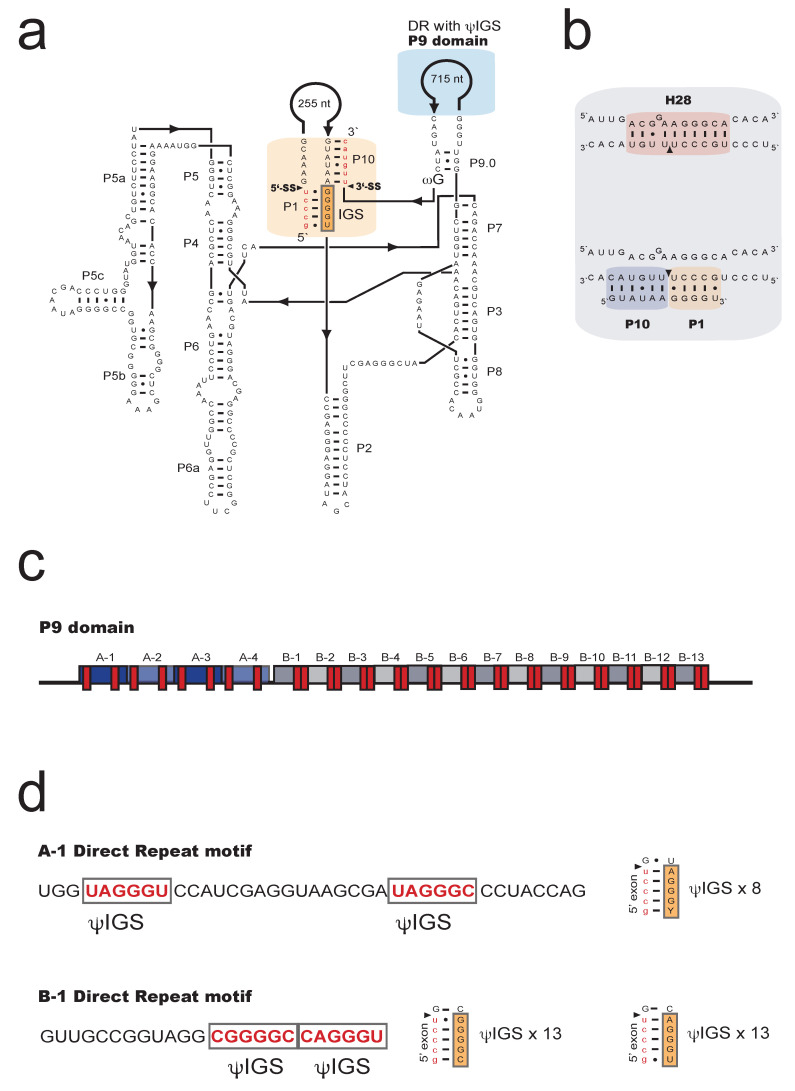
Structural features of the *Mucilago crustacea* S1389 group I intron. (**a**) Secondary structure diagram of the 1274-nt *M. crustacea* S1389 group IC1 intron S1389, presented according to [4]. The intron has a strong P1/P10 exon binding region of 11 base pairs (red box) and a large extension in P9 that contains direct repeat motifs (blue box). P1-P10, paired RNA segments; 5’ SS and 3’SS, exon–intron splice sites; IGS, internal guide sequence. (**b**) Helix 28 (H28) in SSU rRNA becomes completely disrupted when the S1389 intron is present. (**c**) The P9 insertion contains two repeat motifs (A and B) with ΨIGS sequences (red boxes). (**d**) Sequential features of the repeats and putative exon base pair interactions.

Sequencing analysis of the sub-Arctic isolate No-K94 of *M. crustacea* rDNA revealed seven group I introns at conserved insertion sites in the SSU and LSU rRNA genes (Figure 1). Two sites (S788 and S1389) contained introns in the SSU rRNA gene, and five sites (L828, L1926, L1949, L2066, and L2449) in the LSU rRNA gene. The *Mucilago* introns are named according to [23] based on the *E. coli* SSU and LSU rRNAs numbering system. All introns fold into characteristic group I ribozyme core structures (Figure 2a, Supplementary Figure S1a–f), where four belong to the IC1 subgroup (Mcr.S788, Mcr.S1389, Mcr.L828, Mcr.L2449), two to the IE subgroup (Mcr.L1926, Mcr.L2066), and one is unclassified due to a cryptic ribozyme core structure lacking segment P8 (Mcr.L1949). Three *Mucilago* introns (Mcr.L1949, Mcr.S788, and Mcr.S1389) contained direct repeat sequence motifs in peripheral regions of paired segments P1, P2.1, and P9, respectively.

### 2.2. A Complex Group I Intron in M. Crustacea SSU rRNA Helix 28 at Insertion Site S1389

Intron Mcr.S1389 has a complex structural organization at the RNA level (Figure 2a). The 1274 nt intron interrupts the critical neck helix (H28) in the SSU rRNA [24] that connects the body and head of the ribosomal small subunit, and presence of the S1389 intron sequences completely disrupts H28 (Figure 2b). The large size of Mcr.S1389 is mainly due to extension sequences in P1 (255 nt) and P9 (715 nt), but no recognizable open reading frames are present. P9, however, contains a complex array of direct repeats (Figure 2c) consisting of three copies of A-motif (39 nt) and 13 copies of B-motif (24 nt). A closer inspection of motifs A and B identified 34 putative internal guide sequences (ψIGS) (Figure 2d).

### 2.3. Structural Variation among the S1389 Introns

**Figure 3:**
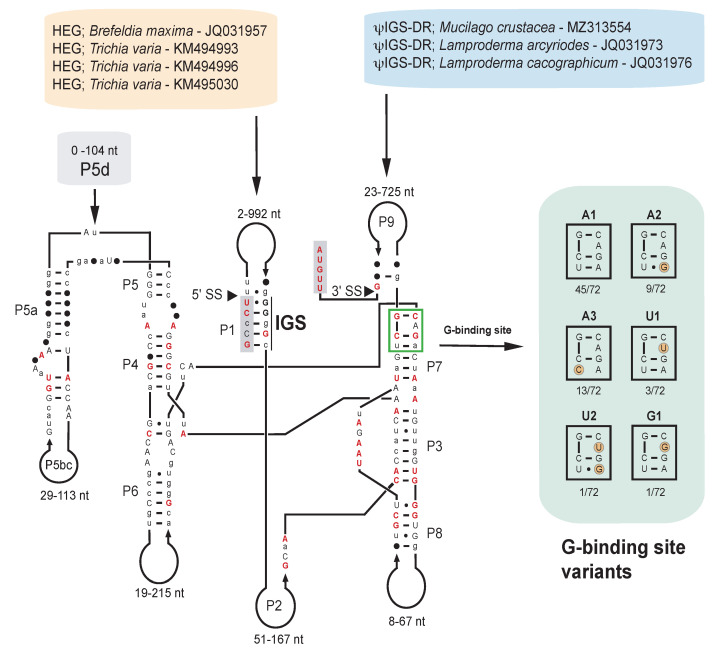
Consensus secondary structure diagram of S1389 group I intron in myxomycetes. The consensus structure is based on 166 nucleotide positions in the catalytic core common among introns from 72 taxa (see Supplementary Table S1). Extensive sequence size variations are noted in most peripheral regions, including the taxa-specific P5d extension. While all homing endonuclease genes (HEGs) are found as P1 extensions, all direct repeat (DR) extensions are located in P9. The latter contains multiple pseudo internal guide sequence (ΨIGS) features. Guanosine binding structure variants in P7 are indicated (boxed at right; G-site). P1-P10, paired RNA segments; 5’ SS and 3’SS, exon–intron splice sites. Invariant nucleotide positions among the 72 introns are shown as red uppercase letters. Black uppercase letter, >90% conservation; lowercase letters, ≥50% conservation; filled circles, <50% conservation.

Group I introns at position S1389 are almost exclusively restricted to myxomycete protists, and introns from 72 taxa representing four orders (Physarales, Stemonitales, Liceales and Trichiales), 15 genera, and 37 species were identified. The majority of the SSU rRNA gene sequences were retrieved from the NCBI database, but six Didymiceae sequences (Supplementary Table S1) were generated in this study. To assess structural variations among the S1389 introns, 166 nucleotide positions in the catalytic core region common to all 72 introns were strictly aligned according to secondary structure features (Supplementary Figure S2). The consensus secondary structure diagram (Figure 3) shows a typical group IC1 intron fold with a conserved catalytic domain (P3-P7-P8) and P4-P5a. Interestingly, six sequence variants of the G-site in segment P7 (A1, A2, A3, U1, U2, and G1) were observed, and named according to the bulged P7 nucleotide residue (corresponding to A263 in the *Tetrahymena* intron) (Figure 3, right panel). Bulged U and G have not been reported previously in naturally occurring nuclear group I introns.

Most sequence variations were found in peripheral regions of segments P1, P2, P5, P6, P8, and P9 (Figure 3). The optional helical segment P5d appears taxa-specific and was common in Stemonitales introns but absent in Trichiales and most Physarales introns (Supplementary Table S1). The P9 extension in three S1389 introns host extensive direct repeat motifs, all with multiple ψIGS. Similar to the *M. crustacea* intron (order Physarales), *Lamproderma arcyriodes* and *Lamproderma cacographicum* (both of order Stemonitales) contain 52 copies and 17 copies, respectively, of ψIGS (Supplementary Figure S3). Finally, four S1389 introns contain homing endonuclease gene (HEG) insertions in P1, represented by three *Trichia varia* (order Trichiales) isolates and one in *Brefeldia maxima* (order Stemonitales) (Figure 3; Supplementary Table S1).

### 2.4. Spliceosomal Introns Interrupt S1389 Group I Intron HEGs

**Figure 4:**
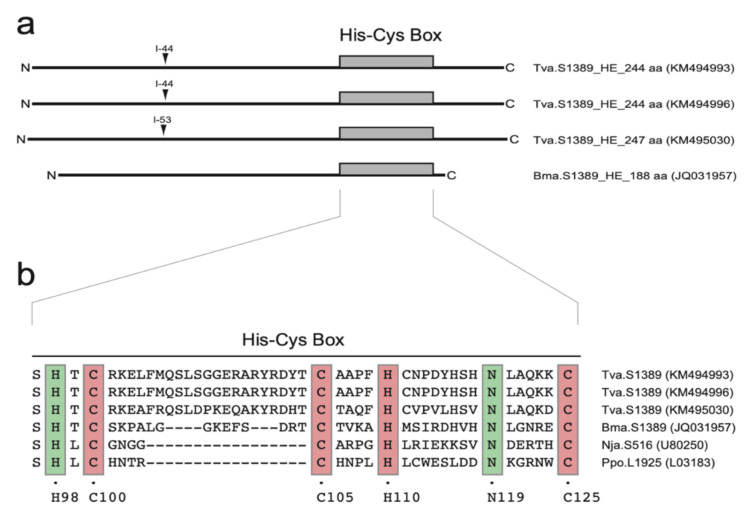
His-Cys homing endonucleases encoded by S1389 group I introns. (**a**) Schematic organization of S1389 intron homing endonucleases including N and C terminal ends, and the characteristic His-Cys box. *Trichia varia* homing endonuclease genes (HEGs) are interrupted by small spliceosomal introns (I-44 and I-53). (**b**) Amino acid alignment of His-Cys box features. The *Naegleria* (I-*Nja*I; Nja.S516) and *Physarum* (I-*Ppo*I; Ppo.L1925) represent well-studied His-Cys homing endonucleases. Conserved residues (boxed) corresponding to those presented in the I-*Ppo*I crystal structure [26]. C100, C105, H110, and C125 are involved in zinc binding, and H98 and N119 are associated with the active site.

The S1389 HEGs were organized in a sense orientation in P1 compared to that of the intron ribozyme and host SSU rRNA and encode His-Cys homing endonuclease proteins of 244 amino acids and 247 amino acids in *T. varia*, and 188 amino acids in *B. maxima* (Figure 4a). Interestingly, the *Trichia* HEGs were found interrupted by small spliceosomal introns of 44 bp and 53 bp (Figure 4a; Supplementary Figure S4). An amino acid sequence alignment of the S1389-derived His-Cys boxes to the well-characterized homing endonucleases in *Naegleria* (I-*Nja*I) [25] and *Physarum* (I-*Ppo*I) [26] (Figure 4b) confirmed that the intron proteins were members of the His-Cys homing endonuclease family.

### 2.5. S1389 Group I Introns Appear Vertically and Horizontally Inherited

S1389 introns were identified in distantly related taxa representing four myxomycete orders (Supplementary Table S1). To gain information about the evolutionary history of S1389 introns, phylogenetic analyses based on group I intron core sequences and on the corresponding host SSU rRNA gene sequences were performed. A phylogenetic tree obtained from maximum likelihood (ML) analysis based on 1575 aligned SSU rRNA positions from 72 taxa is shown in Supplementary Figure S5. The tree topology corrolates well to the current taxonomy of myxomycetes and more recently reported rDNA-based phylogeny [27,28,29,30]. However, we noted that the *Lepidoderma tigrinum* sequence (GenBank accession number DQ903678) appeared embedded within the *Diderma* cluster. Phylogenetic analysis of the corresponding S1389 intron from the same 72 taxa was challenging due to a large dataset and only 166 nt aligned positions. A neighbor-joining tree of the S1389 introns is presented in Supplementary Figure S6. Statistical support was obtained in topologies within several branch clusters, but not well between clusters. Anyway, we observed an agreement between the SSU rDNA and S1389 intron trees, but with some notable exceptions (see below).

**Figure 5:**
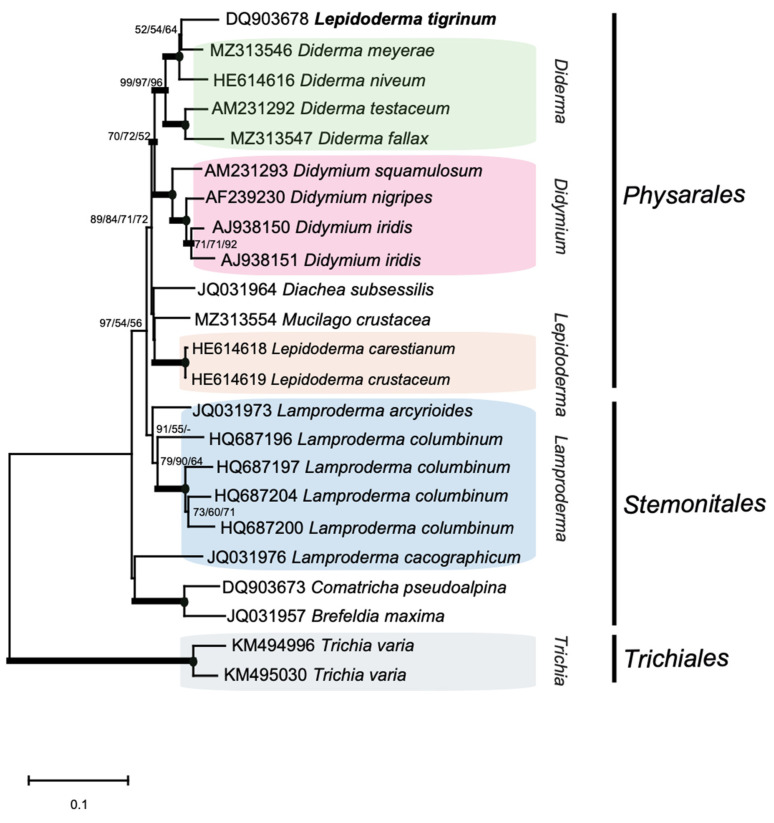
Molecular phylogeny of myxomycete taxa based SSU rDNA sequences. The SSU topology is obtained by neighbor-joining (NJ) analysis of 23 selected taxa and 1575 nt aligned positions (SSU dataset 2; Supplementary Table S1). The tree is rooted with the *T. varia* SSU rDNA sequence. The NJ, maximum likelihood (ML) and maximum parsimony (MP) bootstrap replicates (≥50%) are given at each node. Bayesian posterior (BI) probabilities (≥0.95) are shown in bold branches. Black dots at branch points; maximum support in NJ, ML, and MP (≥97%). Misannotated SSU rDNA in GenBank (*L. tigrinum* DQ903678) is indicated in bold letters. The scale bar indicates the fraction of substitutions per site.

**Figure 6:**
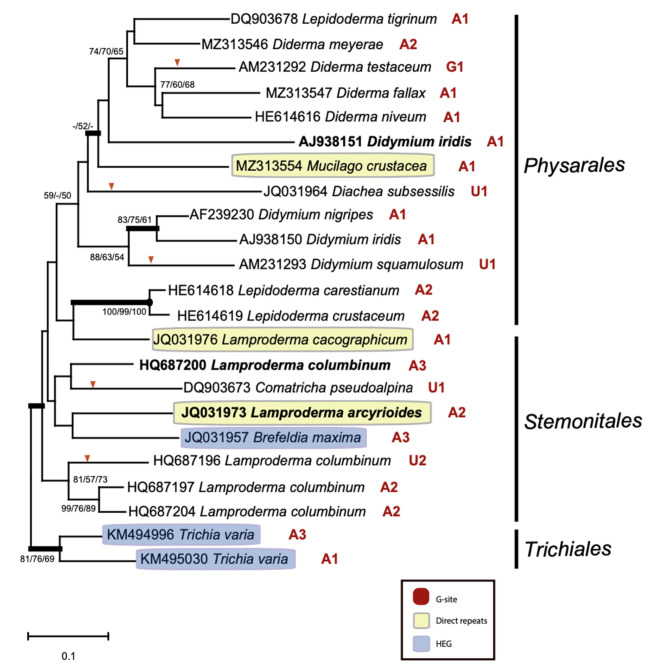
Molecular phylogeny of myxomycete S1389 group I introns. The intron topology is obtained by neighbor-joining (NJ) analysis of 23 taxa and 166 nt aligned positions (intron dataset 2; Supplementary Table S1). The tree is rooted with the *T. varia* S1389 intron sequence. The NJ-, maximum likelihood (ML) and maximum parsimony (MP) bootstrap replicates (≥50%) are given for each node. Bayesian posterior (BI) probabilities (≥0.95) are shown in bold branches. Black dots at branch points; maximum support in NJ, ML, and MP (≥97%). Introns that harbor homing endonuclease genes (HEGs) and direct repeat arrays are indicated by yellow and blue boxes, respectively. G-site variation is indicated at each intron and bulge-nucleotide mutation (A to U; A to G) is indicated on branch by red arrow. Horizontally inherited intron candidates (*D. iridis* AJ938151; *L. columbinum* HQ687200; *L. arcyrioides* JQ031973) are indicated in bold letters. The scale bar indicates the fraction of substitutions per site.

To investigate the distribution of intron structural variants and possible horizontal intron transfer at higher resolution we reduced the selected taxa to 23 in SSU rDNA and corresponding intron phylogenetic analyses. Several interesting and apparently relevant observations were noted when comparing branch patterns of the SSU rDNA-based tree (Figure 5) and the intron-based tree (Figure 6). (1) An overall similarity was found between S1389 introns and their gene hosts (SSU rDNA), suggesting that most S1389 introns possess a vertical inheritance pattern during myxomycete evolution. (2) Three S1389 introns, representing *D. iridis* (AJ938151), *Lamproderma columbinum* (HQ687200), and *L. arcyrioides* JQ031973) appear horizontally inherited. (3) *L. tigrinum* (DQ903678) appears misannotated in GenBank and should most likely be reclassified as a *Diderma* sp. (4) The ψIGS-containing direct repeats in introns from *M. crustacea*, *L. arcyriodes,* and *L. cacographicum* have most likely been independently gained during the evolution. (5) HEGs noted in *B. maxima* and *T. varia* were also independently gained, but we were not able to conclude about evolutionary history of the two *T. varia* HEG variants. (6) The G-site variants (bulge A to U, and bulge A to G mutations) in P7 appear sporadically in closely related S1389 introns. An example is the U1 variant in *D. squamulosum* that appeared generated from the A1 variant by one point mutation (Figure 3 and Figure 6). Similarly, the U2 variant in *L. columbinum* and the G1 variant in *Diderma testaceum* were only one point mutation apart from A2 and A1 variants, respectively (Figure 3 and Figure 6).

## 3. Discussion

We report the nuclear rDNA sequence of the myxomycete *M. crustacea* to contain seven group I introns, which include a complex intron at position S1389 with interesting structural features. A dataset of 72 S1389 introns representing different myxomycete taxa was collected and assessed for structural variation in intron organization and ribozyme core. We identified taxa-specific RNA segments, G-site variants, complex direct repeat arrays, and homing endonuclease genes. The latter contained small spliceosomal introns that have to be removed in order to generate the open reading frames. Phylogenetic reconstruction of intron and host gene suggested an evolutionary history of the S1389 introns that includes both vertical and horizontal intron transfers.

Group I introns are common in myxomycete rDNA, but intron numbers vary between species that have been SSU and LSU rRNA gene sequenced. *P. polycephalum* and *D. iridis*, which are the best studied myxomycete species, carry only 2–3 introns [31,32,33], but *Diderma niveum* rDNA contains more than 20 group I introns [3]. The seven group I introns reported in *M. crustacea* is an intermediate number, but still apparently challenges the pre-rRNA processing. A study of RNA processing and self-splicing in 12 *Fuligo septica* group I introns (4 in the SSU rRNA gene and 8 in the LSU rRNA gene) revealed that each insertion site represents a distinct intron family and that introns were removed in a non-restricted order [34]. The exception, however, was two compulsory introns in the LSU rRNA gene (L1949 and L2449) that spliced more slowly than the others and were the only *F. septica* introns without in vitro self-splicing activities. The L1949 and L2449 introns are compulsory in Physarales myxomycetes [29,35], and present in *M. crustacea*. The truncated catalytic core lacking segment P8 in the *M. crustacea* L1949 intron (Supplementary Figure S1d) was similar to that of *Physarum* [31,36] and *Fuligo* [34] and may in part explain why these introns do not self-splice as naked RNA at standard reaction conditions in vitro.

S1389 introns in myxomycetes constitute a phylogenetically distinct family of nuclear group I introns with a common origin [22], a notion supported by this work. Interestingly, the S1389 introns appeared dependent on essential host splicing factors since they do not self-splice in vitro [22]. However, the ribozyme core structure folding was in agreement with other well-studied group IC1 introns, such as the in vitro self-splicing *Tetrahymena* LSU rRNA intron [37]. This included the organization of the 5’ SS and P1 [38], the A-rich bulges in P5a and P4/P5 [39], and the joining of P4 to the catalytic core [40]. The most surprising structural core variation was at the G-site, which is usually highly conserved among related introns at the same insertion site. Here, a strong covariation has been reported between the P7 bulge nucleotide (corresponding to *Tetrahymena* A263) and the closing base pair (*Tetrahymena* C262:G312) [41,42], forming a conserved base triple [5]. Among the myxomycete S1389 introns we noted six major variants, which included a shift where the A-bulge nucleotide was replaced at U. How this G-site substitution affects catalysis is not known, but a similar sequence variant was obtained by in vitro selection experiments of the *Tetrahymena* intron and found active in self-splicing [43].

The *M. crustacea* S1389 intron harbors a complex direct repeat pattern in peripheral segment P9, and direct repeats are also present in two additional *Mucilago* introns (S788 and L1949). Direct repeat insertions have been reported in several nuclear group I introns in myxomycetes, especially in introns lacking in vitro self-splicing activity. The *F. septica*, *P. flavicomum,* and *D. niveum* L1949 introns contained repeated copies of 45-bp, 101-bp, and 20-bp motifs, respectively, in segment P1 [34,35,36]. Furthermore, the L2449 introns carry direct repeat arrays in several species including *Didymium dubium* that harbored two copies of two different motifs (71 bp and 303 bp) in segment P1 [35], and *Physarum didermoides* with direct repeats in segments P2 (5 copies of 60 bp) and P9 (5 copies of 100 bp) [29]. Functional roles linked to such direct repeat arrays are currently not known. The presence of multiple copies of putative IGS features in the *M. crustacea* S1389 intron and in two additional myxomycete S1389 introns (*L. arcyriodes* and *L. cacographicum*) may suggest a functional role. On the other hand, large extension sequences in segments P1, P2, or P9 do not interfere with group I ribozyme core structures and may be accepted within introns as long as the introns are efficiently removed, probably aided by host-encoded factors. Since direct repeat arrays have sporadic occurrences and probably are evolutionary neutral, they could be considered as non-functional junk RNA similar to what is proposed for the majority of vertebrate long non-coding RNAs [44].

Two myxomycete species, *B. maxima* and *T. varia*, harbor S1389 introns with HEGs in segment P1. These HEGs encode His-Cys-type homing endonucleases typical for nuclear group I introns [3,6] and indicate that S1389 introns may perform intron homing at appropriate biological conditions. Interestingly, the *T. varia* HEGs were interrupted by small spliceosomal introns of 44 bp and 53 bp. Related spliceosomal introns have been reported to splice out and restore functional HEGs in two *D. iridis* group I introns [2,45,46]. A plausible role of the spliceosomal introns may be to facilitate the expression of HEGs located within a rDNA locus by recruiting spliceosomes and probably exon junction complexes [47,48].

## 4. Materials and Methods

### 4.1. Culturing, DNA Isolation, and DNA Sequencing

The myxomycete *M. crustacea* (isolate No-K94) was collected as spores in sub-Arctic Norway (68°N 45′N, 19°N 42′E). Germinated amoebae from *M. crustacea* and some other Physarales myxomycetes (Supplementary Table S1) were grown on DS/2 media [15] at 26 °C, feeding on heat-inactivated *Escherichia coli*. Total DNA was isolated as previously described [34,49], and rDNA was amplified by PCR using various primers designed from known myxomycete rDNA sequences. Sanger sequencing on both strands was performed as previously described [34].

### 4.2. Sequence Alignments

Six of the SSU rDNA sequences, including group I introns, were generated in this work (Supplementary Table S1), and 67 SSU rDNA sequences (including group I introns) were downloaded from the NCBI database (https://www.ncbi.nlm.nih.gov), accessed 1 May 2021). SSU rDNA sequences (with introns removed) were assembled and aligned in the software program Geneious prime^®^ 2019.2.3 (https://www.geneious.com, accessed 1 April 2021). Multiple sequence alignments were generated using MAFFT version 7.450 [50,51] with default setting. The SSU dataset 1 (1575 bp) included sequences from all 72 taxa and was based on published information about myxomycete SSU rRNA [30,52] and evaluated and manually trimmed by excluding parts of variable regions that were not confidently aligned. SSU dataset 2 included 23 taxa and was made similar to dataset 1. Group I intron S1389 core sequences (166 bp) were manually aligned and strictly based on secondary structure features as previously described [22,49] using the Geneious prime^®^ software program. The intron datasets 1 and 2 corresponded to the SSU datasets 1 and 2, except that *T. thermophila* L1925 and *M. crustacea* S788 introns were included as outgroups.

### 4.3. Phylogenetic Analysis

The tree-building methods of neighbor joining (NJ), maximum likelihood (ML), and maximum parsimony (MP) in MEGA X [53] with default settings, as well as Bayesian inference (BI) in MrBayes v.3.2.6 [54], were used in SSU datasets 1 and 2 to construct molecular phylogenetics. All sequence alignments were model tested prior to tree constructions by MEGA X software [53]. The topology of NJ, ML, and MP trees was evaluated by bootstrap analyses. The evolutionary history of SSU dataset 1 was reconstructed with ML using the general time reverse model (GTR) [55] with gamma-distributed rate (G) of heterogeneity among sites approximated by five categories (500 replicates) and MP (subtree-pruning-regrafting algorithm and 500 bootstrap replicates). The phylogeny was also tested by BI conducted with MrBayes v.3.2.6 [54] interpreted in Geneious prime^®^ 2019.2.3. BI was run with default settings with the substitution model, GTR + G (4 categories), including the chain length (1,100,000), subsampling frequency (200), heated chains (4), heated chain temperature (0.2), burn-in length (100,000), and random seed (23,060). The evolutionary history of SSU dataset 2 was reconstructed with NJ with Kimura 2 (K2) evolutionary model [56], ML with GTR + G, MP, and BI. The robustness was tested by bootstrapping (500 replicates).

The evolutionary history of intron dataset 1 was reconstructed with NJ and the Jukes-Cantor (JC) model using MEGA X [53] with default settings. To test the robustness of the nodes, the trees were tested with NJ-JC (500 replicates), MP-SPR (500 replicates), and ML-K2 (500 replicates). BI were conducted with MrBayes v.3.2.7 [57,58] with 10 million generations and sampled every 1000, with a burn-in fraction of 0.25. Intron dataset 2 included 23 taxa. The evolutionary history was reconstructed with NJ and JC model using MEGA X [53] with default settings. The robustness of the nodes of the trees was tested with NJ-JC (500 replicates), MP-SPR (500 replicates), and ML-K2 (500 replicates). BI were conducted with MrBayes v.3.2.7 using the same parameters as in intron dataset 1.

## 5. Conclusions

Myxomycete protists are a frequent source of nuclear group I introns and *M. crustacea* was found to contain seven rDNA group I introns, two in the SSU rRNA gene and five in the LSU rRNA gene. The *M. crustacea* S1389 intron has a complex structural organization and we collected, analyzed, and compared 72 S1389 intron from a wide spectrum of myxomycetes. The S1389 intron sequences and catalytic core structures were clearly related and supported the notion that introns occupying a specific insertion site represent a distinct group I intron family. However, sequence variation at the highly conserved G-site suggested that group I introns may accept more structural changes at the catalytic site than previously anticipated for natural group I ribozymes. Peripheral segments, on the other hand, may harbor large structural insertions containing homing endonuclease genes or direct repeat arrays, RNA sequences that apparently do not interfere with the splicing reaction and the ribozyme core. An interesting and unusual feature was that a second class of introns, the spliceosomal introns, were present within some of the S1389 group I introns. These small introns may have a biological role, probably linked to homing endonuclease gene expression.

## Data Availability

Sequencing data are available in GenBank under the accession numbers MZ313546; MZ313547; MZ313548; MZ313549; MZ313552, and MZ313554.

## Supplementary Materials

The following are available online at https://www.mdpi.com/article/10.3390/ncrna7030043/s1, Figure S1: Secondary structure diagrams of the *Mucilago crustacea* rDNA group I introns, Figure S2: Sequence alignment of core structure nucleotides of myxomycete S1389 group I intron, Figure S3: Secondary structure diagrams of *Lamproderma arcyriodies* and *Lamproderma arcyriodies* S1389 group I intron, Figure S4: Sequence features in *Trichia varia* S1389 group I introns, Figure S5: Molecular phylogeny of myxomycete taxa based SSU rDNA sequences, Figure S6: Molecular phylogeny of myxomycete S1389 group I introns, Table S1: Key features of 72 myxomycete S1389 group I introns included in this work.
