# Supplementary material for: A Phylogenetic Approach to Structural Variation in Organization of Nuclear Group I Introns and Their Ribozymes
Source: Noncoding RNA. 2021 Jul 22;7(3):43. doi: 10.3390/ncrna7030043 (PMC8395846; doi:10.3390/ncrna7030043)
Supplement: Supplementary file 1 [file ncrna-07-00043-s001.zip › ncrna-1280711-supplementary.pdf]

Table S1. Key features of 72 myxomycete S1389 group I introns included in this work.

Host species	Acc No	Isolate	Size [1]	Insert [2]	P5d [3]	P7 [4]
**Order Physarales**						
Diderma fallax [5]	MZ313547	It-K52	532 bp	-	-	A1
D. globosum	DQ903677	AMFD110	722 bp	-	-	A1
D. meyerae	HE614614	It-K61	735 bp	-	-	A2
D. meyerae [5]	MZ313546	It-K68	921 bp	-	-	A2
D. niveum [5]	MZ313548	Fr-M26	749 bp	-	-	A1
D. niveum	AM231291	Fr-K10	682 bp	-	-	A1
D. niveum	HE614616	It-K66	683 bp	-	-	A1
D. niveum	HE614617	Uk-K79	683 bp	-	-	A1
D. saundersii [5]	MZ313549	Mx-K30	532 bp	-	-	A1
D. testeceum	AM231292	Pr3-1	529 bp	-	-	G1
Didymium iridis	AJ938151	CR19-1	523 bp	-	-	A1
D. iridis	AJ938150	CUR1-4	637 bp	-	-	A1
D. nigripes	AF239230	-	654 bp	-	-	A1
D. squamulosum	AM231293	CR10	792 bp	-	-	U1
Fuligo leviderma	DQ903676	AMFD130	517 bp	-	-	A1
Lepidoderma carestianum	HE614618	It-K71	393 bp	-	-	A2
L. crustaceum	HE614619	It-K62	396 bp	-	-	A2
L. peyerimhoffii [5]	MZ313552	It-K63	388 bp	-	+	A2
L. tigrinum	DQ903678	AMFD192	785 bp	-	-	A1
Mucilago crustacea [5]	MZ313554	No-K94	1274 bp	DR-P9	-	A1
**Order Stemonitales**						
Brefeldia maxima	JQ031957	MM24519	1304 bp	HEG-P1	+	A3
Colloderma oculatum	JQ031959	HS2885	421 bp	-	+	A3
C. robustum	JQ031960	AMFD270	384 bp	-	+	A1
Comatricha nigra	DQ903683	AMFD155	788 bp	-	+	A1
C. pseudoalpina	DQ903673	MM23892	815 bp	-	+	U1
Diacheopsis pauxilla	JQ031966	MM29883	628 bp	-	-	A1
Diachea subsessilis	JQ031964	MM24463	919 bp	-	-	U1
Lamproderma aeneum	JQ031969	MM36255	545 bp	-	-	A3
L. arcyrioides	JQ031973	MM37005	1006 bp	DR-P9	+	A2
L. cacographicum	JQ031976	AMFD310	957 bp	DR-P9	-	A1
L. columbinum	HQ687204	F2	503 bp	-	-	A2
L. columbinum	HQ687196	106	563 bp	-	-	U2
L. columbinum	HQ687197	63b	474 bp	-	-	A2
L. columbinum	HQ687200	132	480 bp	-	+	A3
L. disseminatum	JQ031978	AMFD38	498 bp	-	-	A1
L. echinosporum	JQ031980	AMFD136	486 bp	-	+	A3
L. echinosporum	JQ031979	AK06016	481 bp	-	+	A3
L. pseudomaculatum	JQ031985	MM37354	411 bp	-	-	A3
L. puncticulatum	HQ687194	172	514 bp	-	+	A2
L. puncticulatum	HQ687202	162	511 bp	-	+	A1
L. puncticulatum	HQ687195	3	517 bp	-	+	A1
L. sauteri	DQ903674	AMFD208	543 bp	-	+	A1
L. zonatum	DQ903672	MM21644	434 bp	-	-	A1
Meriderma carestiae	JQ031999	MM35985	566 bp	-	-	A1
M. carestiae	DQ903671	AMFD173	566 bp	-	-	A1
M. cribrarioides	JQ032000	MM37106	538 bp	-	-	A1
**Order Liceales**						
Licea marginata	JX481296	DWM7368	455 bp	-	+	A3
**Order Trichiales**						
Calomyxa metallica	JX481284	AMFD483	532 bp	-	-	A3
Trichia varia	KM494993	sc22370	1531 bp	HEG-P1	-	A3
T. varia	KM494994	LE259268	586 bp	-	-	A3
T. varia	KM494995	LE259461	586 bp	-	-	A3
T. varia	KM494996	JVR848	1531 bp	HEG-P1	-	A3
T. varia	KM494997	sc22386	559 bp	-	-	A1
T. varia	KM494998	sc22408	559 bp	-	-	A1
T. varia	KM494999	sc22409	559 bp	-	-	A1
T. varia	KM495003	sc22442	559 bp	-	-	A1
T. varia	KM495005	sc22517	559 bp	-	-	A1
T. varia	KM495006	sc27697	559 bp	-	-	A1

T. varia	KM495009	sc27742	559 bp	-	-	A1
T. varia	KM495010	sc27839	559 bp	-	-	A1
T. varia	KM495018	KRAM M-1585	559 bp	-	-	A1
T. varia	KM495019	sc27686	559 bp	-	-	A1
T. varia	KM495020	sc27737	559 bp	-	-	A1
T. varia	KM495021	sc27850c1	559 bp	-	-	A1
T. varia	KM495022	sc27860c4	559 bp	-	-	A1
T. varia	KM495023	sc27507	559 bp	-	-	A1
T. varia	KM495024	sc27648c1	559 bp	-	-	A1
T. varia	KM495026	sc27667c1	559 bp	-	-	A1
T. varia	KM495027	sc27667c2	559 bp	-	-	A1
T. varia	KM495028	sc27772c2	559 bp	-	-	A1
T. varia	KM495029	sc27850c2	559 bo	-	-	A1
T. varia	KM495030	LE254838	1544 bp	HEG-P1	-	A1
**Host species**	**Acc No**	**Isolate**	**Size**	**Insert**	**P5d**	**P7**

Notes:

[1] Size in base pairs of the S1389 group I intron insertion.

[2] Type of large insertions in group I ribozyme paired segment P1 or P9. HEG, homing endonuclease gene; DR, direct repeat motifs.

[3] Presence or absence of the optional paired segment P5d.

[4] G-binding sequence variants in segment P7, as indicated in Figure 3.

[5] This work.

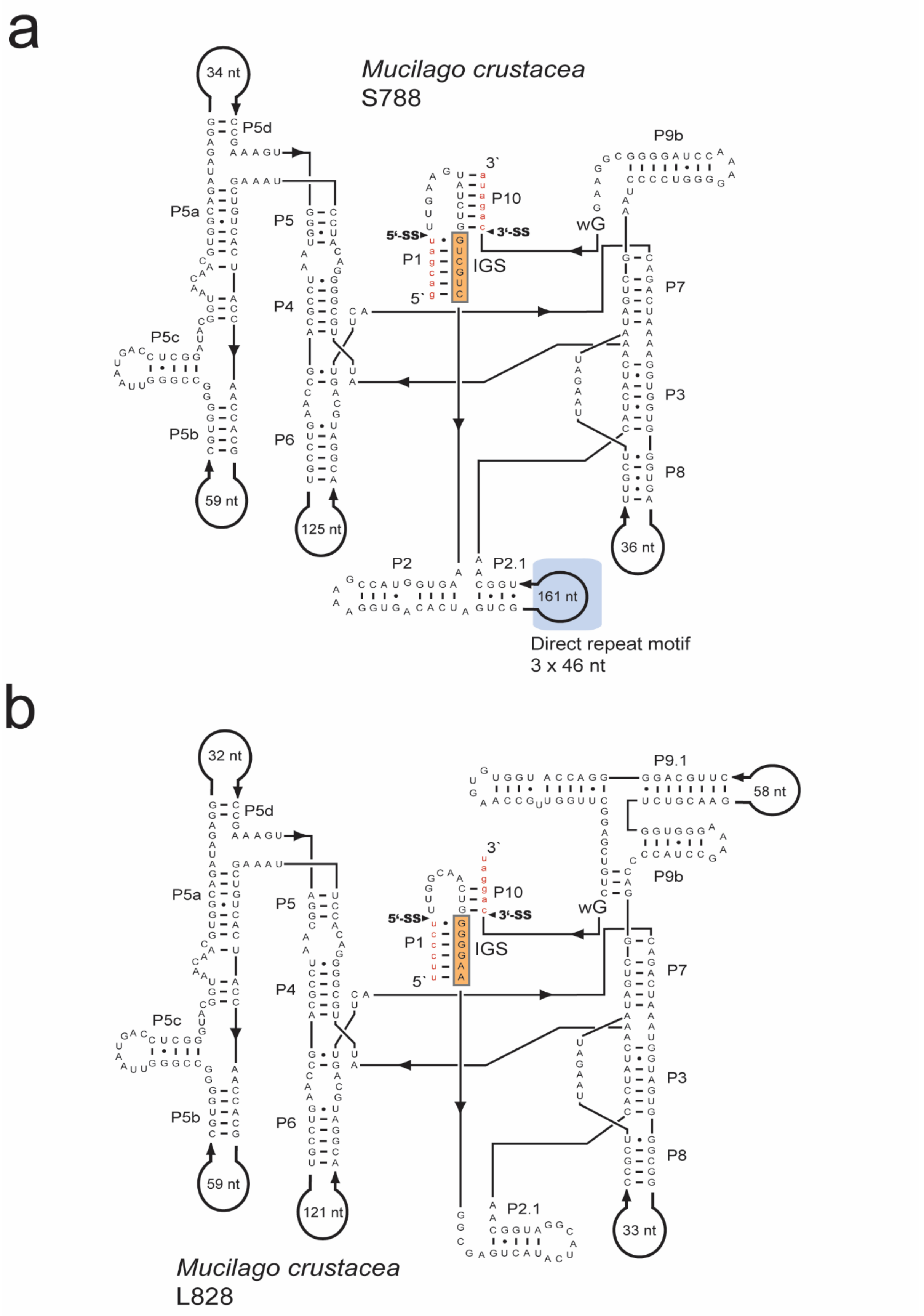
a
Mucilago crustacea
S788
34 nt
P5d
P5a
P5
P5c
P4
P5b
P6
59 nt
125 nt
P9b
3`
P10
5'-SS
3'-SS
wG
P1
IGS
5`
P7
P3
P8
36 nt
P2
P2.1
161 nt
Direct repeat motif
3 x 46 nt
b
32 nt
P5d
P9.1
58 nt
P9b
3`
P10
5'-SS
3'-SS
wG
P1
IGS
5`
P5a
P5
P4
P5c
P7
P3
P5b
P6
P8
59 nt
121 nt
P2.1
33 nt
Mucilago crustacea
L828

Figure S1 (a&b)

c

Mucilago crustacea
L1926

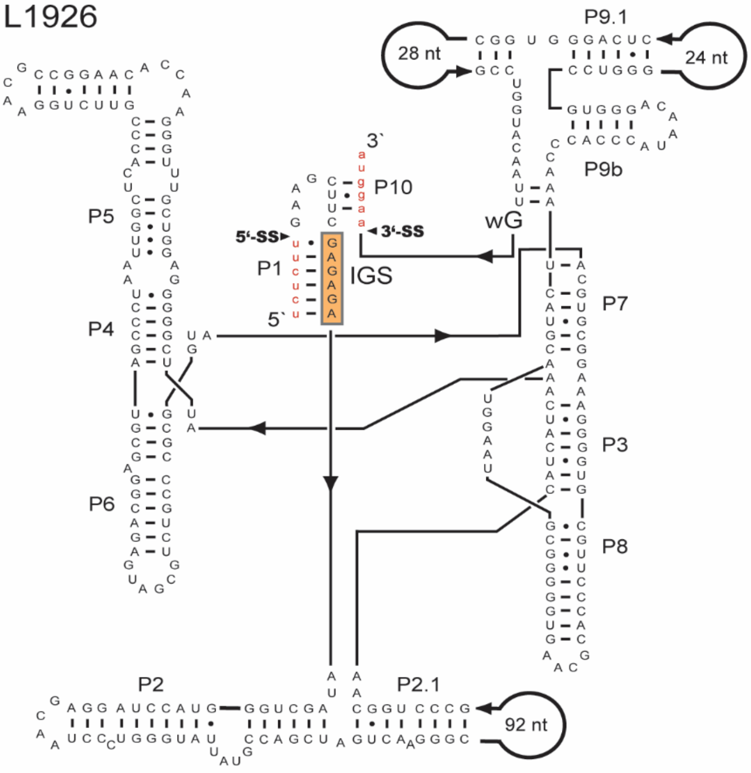

d

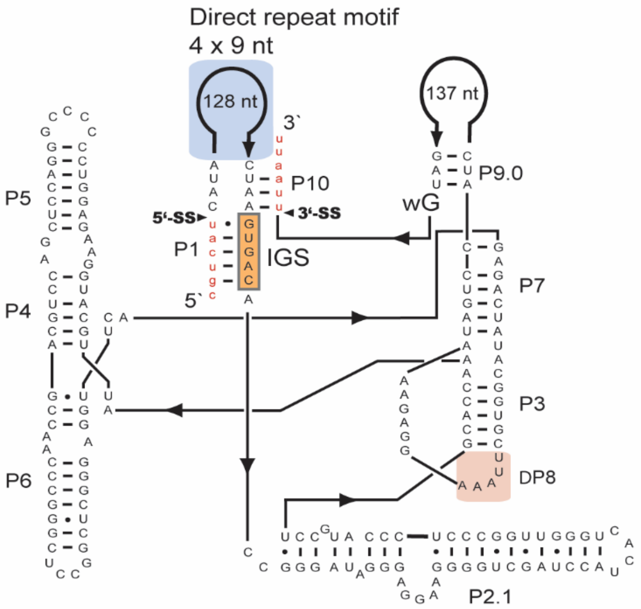

Mucilago crustacea
L1949

Figure S1 (c&d)

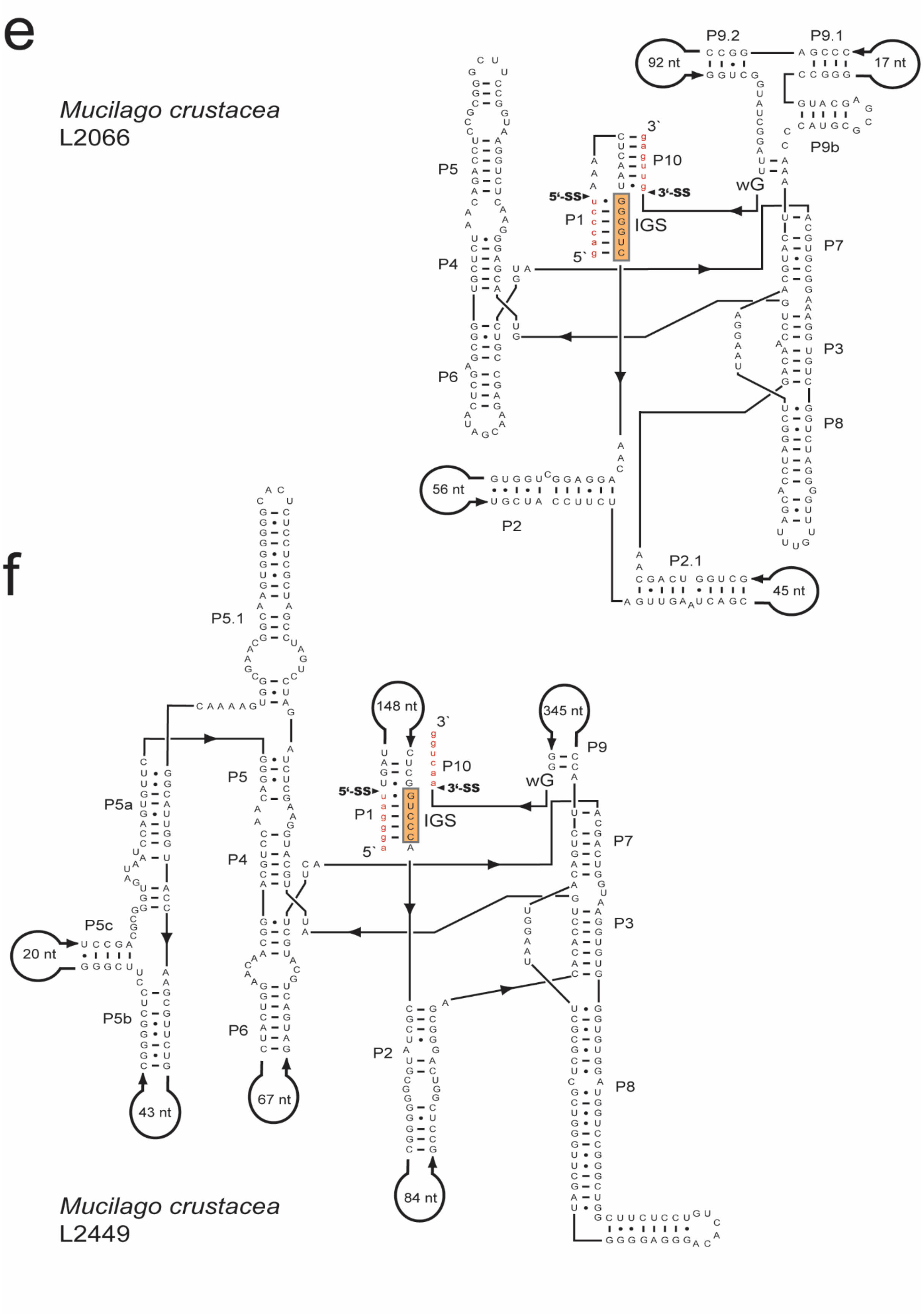

Figure S1 (e&f)

Figure S1: Secondary structure diagrams of the *Mucilago crustacea* rDNA group I introns. (**a**) S788 is a group IC1 intron in the SSU rRNA gene. Note that segment P2.1 (blue box) contains a 46-nt direct repeat motif. (**b**) L828 is a group IC1 intron in

the LSU rRNA gene. (**c**) L1926 is a group IE intron in the LSU rRNA gene. (**d**) L1949 is an unclassified group I intron in the LSU rRNA gene. Note that the P8 segment of the catalytic core is missing (pink box), and that segment P1 (blue box) contains a 9-nt direct repeat motif. (**e**) L2066 is a group IE intron in the LSU rRNA gene. (**f**) L2449 is a group IC1 intron in the LSU rRNA gene. Note that P1 and P9 contain extension sequences, but with no reading frame or direct repeat features. Common note to all intron diagrams: P1-P10, paired RNA segments; 5' SS and 3'SS, exon-intron splice sites; IGS, internal guide sequence; ωG, last nucleotide residue of intron; red lowercase letters, exon sequences.

```
                  <<P1>>>  <<P1>    <<P3>>>    <<<P4>>>   <P5>>          <<<<P5a>>>>>>      <<<<P5a>>>>>>>>>>>>
MZ313554          gcccuGAAAGGGGUGCUACACUG-ACAAAUU-G--CGGGAAAGGCUCGGUAAAAGGAA-GGCA-CACCAAGUAUGGUAACAGC-UGUC-U-UCCUAU
MZ313547          guccuGAAAGGGGCGCGACACCG-ACAAAUA-G--CGGGGAAUGCCU--UGAA-AGCA-UCGGAUACCAAGUACGGUAGAACG-CCGA-UAGC--AU
MZ313549          gcccuGAAAGGGGCGCGACACCG-ACAAAUA-G--CGGGGAAUGCCU--UGAA-AGCA-UCGGAUACCAAGUACGGUAGAACG-CCGA-UAGC--AU
MZ313548          gcccuGAAAGGGGCGCAACACUA-ACCAAGUAG--CGGGAAUCCUCU--UAAA-AGCA-CUGGAUACCAAGUAUGGUGAUAAAGCCAG-GAGC--AU
MZ313546          gcccuUAAAGGGGCGCAACACUA-ACCAAGUAG--CGGGAAUCUCCU--UAAA-AGCA-CUGGAUACCAAGUAUGGUGAUAAAGCCAG-GCGC--AU
DQ903677          gcccuGAAAGGGGCGCGACACUA-ACCAAGUAG--CGGGAACUCUCCU-UAAA-AGCA-CUGGAUACCAAGUAUGGUGAUAAAGCCAG-UUGC--AU
HE614614          gcccuGAAAGGGGCGCAACACUA-ACCAAGUAG--CGGGAAUCUCCU--UAAA-AGCA-CUGGAUACCAAGUAUGGUGAUAAAGCCAG-GCGC--AU
AM231291          gcccuGAUAGGGGUGCGACACUA-ACAAAUA-G--CGGGGAGUCUCAC-GAAU-AGCA-UCGGCUACCAAGUACGGUAACAACGCCAG-U-GU-AAC
HE614616          gcccuGAUAGGGGUGCGACACUA-ACAAAUA-G--CGGGGAGUCUCAC-GAAU-AGCA-UCGGCUACCAAGUACGGUAACAACGCCAG-U-GU-AAC
HE614617          gcccuGAUAGGGGUGCGACACUA-ACAAAUA-G--CGGGGAGUCUCAC-GAAU-AGCA-UCGGCUACCAAGUACGGUAACAACGCCAG-U-GG-AAU
AM231292          gcccuGCAAGGGGCGCGACACUA-GCAAAUA-G--CGGGAA-GUACC--UAAA-AGCA-CGGGAUACCAAGUACGGUAACAAUGCCCG-UAGC--AU
AJ938151          gcccuACAGGGGGCGCAACAUUG-GUAAAUC-G--CGGUGA-CUCCA--UCUU--GCAUAGGA-UACCAAGUAUGGUCACAAC-UCCU-U-GC--AU
AJ938150          gcccuUUAGGGGGCGUAACAUUG-GCAAAAU-G--CGGGAAAGCCCACCAACCAGCUCUCAUC-UCCCAAGUACGGUAAUAAC-GGUG-U-CG--UA
AF239230          gcccuUCAGGGGGCGCAACACCG-GCAAAAU-G--CGGGGAAGUCCAUCAACCAGCUCUCAUC-UACCAAGUAUGGUAACAAC-GGUG-U-CG-UAG
AM231293          gcccuUCAGGGGGCGCAACACCA-GCAAACU-G--CGGGAACGUCCCUACGCGACUCC-CACC-UACCAAGUACGGUAACAAC-GGUG-G-GGUAGC
HE614618          gcccuUGAGGGGGCGCAACACCG-ACAAAUU-G--CGGGGA-CUCCC--UUAA-GCUG-GCCC-UACCAAGUACGGUAACAAU-GGGC-C-AG--AU
HE614619          gcccuUGAGGGGGCGCAACACCG-ACAAAUU-G--CGGGGACUCCC---UUAGAGCUG-GCCC-UACCAAGUACGGUAACAAU-GGGC-C-AG--AU
MZ313552          gcccuUGAAGGGGCGCAACACCG-ACAAAUU-G--CGGGGACUCCC---UGAA-GCUG-GCCC-UACCAAGUACGGUAACAAU-GGGCUC-AG--AU
DQ903678          gcccuAAAAGGGGCGCGACACUG-ACAAAGUAG--CGGGAAUCCUCU--CAAA-AGUU-CUGGAUACCAAUCCGGGUAACAAC-CGGG-G-GU--AU
DQ903676          gcccuUGUGGGGGCGCGACACUG-UCAAAUU-G--CGGGAACACCC---UAAA-GCUA-UCAC-UACUACGCAUAGUAAUAAC-GUGA-U-AG--AU
JQ031957          gcccuUUUGGGGGCGCGACACUG-CCAAAUU-GAUCGGGAA-CCCCC--UAAA-GAUU-CAGC-UACCAAGUACGGUAAUAAU-GCUG-A-AU--AU
JQ031959          gcccuUGUGGGGGCGCGACACUA-UCAAAUU-G--CAGGAACACCC---UAAA-GACC-CAAC-UACCAAGUACGGUAAUAAC-GUUG-G-GU--AU
JQ031960          gcccuCCAGGGGGCGCGACACUA-CCAAAUU-G--CGGGGAAGCCC---UUAA-GCCC-AGACCUACCAAGUAUGGUAACAACGGUCU-G-GG--AU
DQ903683          gcccuGACAGGGGCGCAACAUUG-UUCAACA-G--CAGGAACGCUCU--CAAA-GGCC-GGCU-CACCCAGGCGAGUAACAGC-UUUU-G-GG--AU
DQ903673          gcccuUGAAGGGGCGCGACACUA-CCAAAUU-G--CGGGAAAACCC---UAAAGGCUU-GCAC-UACCAAGUACGGUGAUAAC-GUGC-A-AG-CAU
JQ031966          gcccuUUAGGGGGCGCGACACUG-ACAAAUU-G--CGGGGACACCC---UUAGAGCCG-GCCC-UACCAAGUAUGGUAACAAU-GGGC-A-GG--AU
JQ031964          gcccuUAUGGGGGCGCGACACUGAGCAAAUC-A--CAGGGA-GUCCUUGCUAUCGACG-AGUC-UACCAAGUACGGUGACAAC-GGCUUC-GU-CGG
JQ031969          gcccuUUAAGGGGCGCAACACUG-ACAAAUU-GA-CGGGGA-GUCCCU-UAGA-GCCC-AUCC-UACCAAGUACGGUAAAAAA-GGAU-G-GG--AU
JQ031973          gcccuGGUGGGGGCGCAACACCA-UCAAAUU-GAUCAGGGA-CCCCC--UAAA-GGCU-CAGC-CACCAAGUACGGUAAUAGU-GCUG-A-GU--AU
JQ031976          gcccuUGUAGGGGUGCAACACUA-ACAAAUU-G--CGGGGA-GUCCCU-UUAGAGCUA-GGCCAUACCAAGUACGGUAAUAACUGGUC-U-GG--AU
HQ687204          gcccuGUAGGGGGCGCGACACCA-UCAAACU-GA-CGGGGA-AUCCCU-UAGA-GCUU-AACC-UACCAAGUACGGUAAAAAA-GGGU-A-AG--AU
HQ687196          gcccuGAAAGGGGCGCAACACCU-UCAAAUU-G--CAGGGA-GUCCCU-UAGA-GCUU-AAACCUACCAAGUACGGUAAAAAUGGUUU-A-AG--AU
HQ687197          gcccuUUAAGGGGCGCAACACCA-CCAAACU-GA-CGGGGA-GUCCCU-UAGA-GCUU-AACC-UACCAAGUAUGGUAAAAAA-GGUUUA-AG--AU
HQ687200          gcccuUGAAGGGGCGCGACACUG-UCAAAUU-GUUCGGGAACACCC---UAAA-GCCU-CCAC-UACCACGCAUGGUAAUAAC-GUGGAA-GG--AU
JQ031978          gcccuCUUAGGGGCGCAACACUG-CCAAAUU-G--CGGGGA-GUCCC--UUAA-GCUU-AGCC-UACCAAGUACGGUAAAAAC-GGUU-A-AG--AU
JQ031980          gcccuUGCGGGGGUGCAACACUG-UCAAAUU-GAUCAGGAACCUCC---UAAA-GCCU-GCAC-UACCACGCAUGGUAAGAAC-GUGU-G-GG--AU
JQ031979          gcccuCCCGGGGGUGCAACACUG-UCAAAUU-GAUCAGGGACCUCC---UAAA-GCCU-GCAC-UACCACGCAUGGUAAGAAC-GUGU-G-GG--AU
JQ031985          gcccuUUAAGGGGUGCGACACUG-ACAAAUU-G--CGGGGA-CUCCCUAUAGA-GCCG-UCCC-UACUAAGUAUAGUAACAAU-GGGA-U-GG-GAU
HQ687194          gcccuAUGAGGGGUGCAACACCA-UCAAAUU-G--CGGGGAACCCAC--UAAC-GGGU-CUGGCUACCAAGAUGGGUUACAAUGCUGG-A-UC--AU
HQ687202          gcccuUCCCGUCGCGCAACACCA-UCAAAUU-G--CGGGAAACCCUU--AGGU-GGGU-CUGGCUACCAAGACAGGUUACAACGCUGG-A-UC--AU
HQ687195          gcccuUCGAGGGGCGCAACACCA-UCAAAUU-G--CGGGGAACCCAC--UAAU-GGGU-CUGGCUACCAAGAUGGGUUACAACGCUGG-A-UC--AU
DQ903674          gcccuUGAAGGGGCGCGACACUA-CCAAAUU-G--CGGGAACCCCC---UAAA-GAUU-UCAGCUACUAAGUACAGUAACAACGCUGU-C-GU--AU
DQ903672          gcccuUGCUGGGGUGCGACACUG-ACAAAUU-GACCGGGAA-UCCCU--UAGA-GCUG-GCCC-UACCAAGUACGGUAAAAAA-GGGU-C-AG--AU
JQ031999          gcccuCUAGGGGGUGCAACACUA-UCAAAUU-G--CGGGGAAGCCCUGUCACGAGCUU-GAUC-UACCAAGUACGGUAACAAC-GAUC-A-GGCCAG
DQ903671          gcccuCUAGGGGGUGCAACACUA-UCAAAUU-G--CGGGGAAGCCCUGUCACGAGCUU-GAUC-UACCAAGUACGGUAACAAC-GAUC-A-GGCCAG
JQ032000          gcccuCUAGGGGGUGCAACACUA-CCAAAUU-G--CGGGGAAGCCCAA-UACAAGCUUAGGCC-UACCAAGUAUGGUAAAAAC-GGUC-U-AG--GU
JX481296          gcucuCUGGGGAGCGCGACACCA-ACCAAUU-G--CGGGGACACCC---UAGC-AGGUAAGCG-CGCCAAGUACGGCAACAGU-CGCG-A-CCUCCU
KM494993          gcucuUUUGGGAGCGCAACACCG-UCAAAUU-GAUCGGGAAGUCCCU--UAGA-GCCU-GCAC-UACCAAGUAUGGUAACAAC-GUGC-A-GG--AU
KM494994          gcucuCUUGGGAGCGCAACACCG-UCAAAUU-GAUCGGGAAGUCCCU--UAGA-GCCU-GCAC-UACCAAGUAUGGUAACAAC-GUGCUA-GG--AU
KM494995          gcucuCUUGGGAGCGCAACACCG-UCAAAUU-GAUCGGGAAGUCCCU--UAGA-GCCU-GCAC-UACCAAGUAUGGUAACAAC-GUGCUA-GG--AU
KM494996          gcucuUUUGGGAGCGCAACACCG-UCAAAUU-GAUCGGGAAGUCCCU--UAGA-GCCU-GCAC-UACCAAGUAUGGUAACAAC-GUGC-A-GG--AU
KM494997          gcucuUUUGGGAGCGCAACACCA-UCAAAUU-GC-CGGGAAGUCCCU--UAGA-GCCC-AUCC-UACCAAGUACGGUAAAAAAGGGAU-G-GG--AU
KM494998          gcucuUUUGGGAGCGCAACACCA-UCAAAUU-GC-CGGGAAGUCCCU--UAGA-GCCC-AUCC-UACCAAGUACGGUAAAAAA-GGAU-G-GG--AU
KM494999          gcucuUUUGGGAGCGCAACACCA-UCAAAUU-GC-CGGGAAGUCCCU--UAGA-GCCC-AUCC-UACCAAGUACGGUAAAAAA-GGAU-G-GG--AU
KM495003          gcucuUUUGGGAGCGCAACACCA-UCAAAUU-GC-CGGGAAGUCCCU--UAGA-GCCC-AUCC-UACCAAGUACGGUAAAAAA-GGAU-G-GG--AU
KM495005          gcucuUUUGGGAGCGCAACACCA-UCAAAUU-GC-CGGGAAGUCCCU--UAGA-GCCC-AUCC-UACCAAGUACGGUAAAAAA-GGAU-G-GG--AU
KM495006          gcucuUUUGGGAGCGCAACACCA-UCAAAUU-GC-CGGGAAGUCCCU--UAGA-GCCC-AUCC-UACCAAGUACGGUAAAAAA-GGAU-G-GG--AU
KM495009          gcucuUUUGGGAGCGCAACACCA-UCAAAUU-GC-CGGGAAGUCCCU--UAGA-GCCC-AUCC-UACCAAGUACGGUAAAAAA-GGAU-G-GG--AU
KM495010          gcucuUUUGGGAGCGCAACACCA-UCAAAUU-GC-CGGGAAGUCCCU--UAGA-GCCC-AUCC-UACCAAGUACGGUAAAAAA-GGAU-G-GG--AU
KM495018          gcucuUUUGGGAGCGCAACACCA-UCAAAUU-GC-CGGGAAGUCCCU--UAGA-GCCC-AUCC-UACCAAGUACGGUAAAAAA-GGAU-G-GG--AU
KM495019          gcucuUUUGGGAGCGCAACACCA-UCAAAUU-GC-CGGGAAGUCCCU--UAGA-GCCC-AUCC-UACCAAGUACGGUAAAAAA-GGAU-G-GG--AU
KM495020          gcucuUUUGGGAGCGCAACACCA-UCAAAUU-GC-CGGGAAGUCCCU--UAGA-GCCC-AUCC-UACCAAGUACGGUAAAAAA-GGAU-G-GG--AU
KM495021          gcucuUUUGGGAGCGCAACACCA-UCAAAUU-GC-CGGGAAGUCCCU--UAGA-GCCC-AUCC-UACCAAGUACGGUAAAAAA-GGAU-G-GG--AU
KM495022          gcucuUUUGGGAGCGCAACACCA-UCAAAUU-GC-CGGGAAGUCCCU--UAGA-GCCC-AUCC-UACCAAGUACGGUAAAAAA-GGAU-G-GG--AU
KM495023          gcucuUUUGGGAGCGCAACACCA-UCAAAUU-GC-CGGGAAGUCCCU--UAGA-GCCC-AUCC-UACCAAGUACGGUAAAAAA-GGAU-G-GG--AU
KM495024          gcucuUUUGGGAGCGCAACACCA-UCAAAUU-GC-CGGGAAGUCCCU--UAGA-GCCC-AUCC-UACCAAGUACGGUAAAAAA-GGAU-G-GG--AU
KM495026          gcucuUUUGGGAGCGCAACACCA-UCAAAUU-GC-CGGGAAGUCCCU--UAGA-GCCC-AUCC-UACCAAGUACGGUAAAAAA-GGAU-G-GG--AU
KM495027          gcucuUUUGGGAGCGCAACACCA-UCAAAUU-GC-CGGGAAGUCCCU--UAGA-GCCC-AUCC-UACCAAGUACGGUAAAAAA-GGAU-G-GG--AU
KM495028          gcucuUUUGGGAGCGCAACACCA-UCAAAUU-GC-CGGGAAGUCCCU--UAGA-GCCC-AUCC-UACCAAGUACGGUAAAAAA-GGAU-G-GG--AU
KM495029          gcucuUUUGGGAGCGCAACACCA-UCAAAUU-GC-CGGGAAGUCCCU--UAGA-GCCC-AUCC-UACCAAGUACGGUAAAAAA-GGAU-G-GG--AU
KM495030          gcucuUUUGGGAGCGCAACACCA-UCAAAUU-GC-CGGGAAGUCCCU--UAGA-GCCC-AUCC-UACCAAGUACGGUAAAAAA-GGAU-G-GG--AU
JX481284          gcucuUUUGGGAGCGCAACACCC-UCAAAUU-GU-CGGGAAGUCCCU--UAGA-GCCC-UCUC-UACCAAGUACGGUAAAAAA-GAGG-G-GGGAGG
V01416            ucucuAAUUGGAGGGCAAGACCG-UCAAAUU-G--CGGGAAAGGGGUC-AACA-GCCGUUCAG-UACCAAGUAUGGUAAUAAG-CUGA-C-GG--AC
MZ313554*         acgauUUUGGUCUGGCAACAUCA-UCAAAUU-G--CGGGGACAUCC---UAAA-GCUG-UCAC-UACCAAAUACGGUAACAAC-GUGG-C-AG--AU
```

```
                         <<P5>  <<<P4>>><<<P6>>>>>><<<P6>>>>>>   <<<P7>>   <<P3>>><<P8><P8>>       <<P7>>    *
MZ313554                 GGUC-AACU-CGCAGCCAAGUCCCUAGGGAUGCAGUUCACAGACCAAACGUCAGUGGGUGGCCGCUUAAGAGAUGGUCGGGUCGuugua
MZ313547                 GGUACGACC-CGCGGCCAAGCCCGUCAGGGUGCAGCCCACAGACCAAACGUUGGUGGGUGGGUGCAUAAGACAUGGUCGUAUCGuugua
MZ313549                 GGUACGACC-UGCGGCCAAGCCCGUCAGGGUGCAGCCCACAGACCAAACGUUGGUGGGUGGCUGCAUAAGACAUGGUCGUAUCGuugua
MZ313548                 GGGC-AACC-UGCGGCCAAGCCCGUCAGGGUGCAGCUCACAGACCAAACGUUAGUGGGUGGCUGCUUAAGAUAUGGUCGACCAGuugua
Mz313546                 GGGGCAACC-UGCAGCCAAGCCCGUUAGGGUGCAGCUCACAGGCCAAACGGUGGUGGGUGGCUGCUUAAGAUAUGGUCGAACCGuugua
DQ903677                 GGGC-AACC-UGCGGCCAAGCCCGUCAGGGUGCAGCUCACAGACCAAACGUUAGUGGGUGGCUGCUUAAGAUAUGGUCGACCCGuugua
HE614614                 GGGGCAACC-UGCAGCCAAGCCCGUCAGGGUGCAGCUCACAGGCCAAACGGUGGUGGGUGGCUGCUUAAGAUAUGGUCGACCCGuugua
AM231291                 GGGAUAACC-UGCGGCCAAGCCCGUCAGGGUGCAGCUACCAGAUCAAACGUUAGUGGGUGAUUGCUUAAGAUAUGAUCGAAUUGuugua
HE614616                 GGGAUAACC-UGCGGCCAAGCCCGUCAGGGUGCAGCUACCAGAUCAAACGUUAGUGGGUGAUUGCUUAAGAUAUGAUCGAAUUGuugua
HE614617                 GGGAUAACC-UGCGGCCAAGCCCGUCAGGGUGCAGCUACCAGAUCAAACGUUAGUGGGUGAUUGCUUAAGAUAUGAUCGAAUUGuugua
AM231292                 GUGU-AACC-UGCGGCCAACCCCGUCAGGGUGCAGCCCACGGACCAAAUCUUAGUGGGUGAUUGCUUAAGAUAUGGUCGCCUCGuugua
AJ938151                 GGUCUAACU-UGCCUCCAAACCCUGUUGGGCUCAGUUCACAGAUCAUACGCUAGUGGGUGGCUGCUUAAGAUUUGAUCGGUUCGuugua
AJ938150                 GGGC-UACU-UGCAACCAAGCCCUGUUGGGUACAGUUCACAGACCAUACGCCAGUGGGUGGCUGCAUAAGACAUGGUCGCGACGuugua
AF239230                 GGGC-AACC-UGCAGCCAAUCCCUUAUGGGUACAGUUCACAGACCAAACGCCGGUGGGUGGCUGCAUAAGACAUGGUCGUAACGuugua
AM231293                 GGAU-UACU-UGCAGCCAAACCCUCGUGGGUCCAGUUCACUGACUAGAAGCUGGUGGGUGGUCGCCUAAGAUGUAGUCGCAACGuugua
HE614618                 GGGU-UAUC-UGCAGCUAACCCCUUACGGGCGUAGUUCACAGGCUAGACGCCGGUGGGUGGCUGCUUAAGAGAUAGUCGUGCAGuugua
HE614619                 GGGU-UAUC-UGCAGCUAACCCCUUACGGGCGUAGUUCACAGGCUAGAUGCCGGUGGGUGGCUGCUUAAGAUAUAGUCGUGGGGuugua
MZ313552                 GGGU-UAUC-CGCAGCUAACCCCUUAUGGGCGUAGUUCACAGGCUAAACGCCGGUGGGUGGCUGCUUAAGAUAUGGUCGUCGAGuugua
DQ903678                 GGGC-AACC-UGCGGCCAAGCCCGUUAGGGUGCAGCUAACAGACCAAACGUCAGUGGGUGGGUGCUUAAGAUAUGGUCGAGACGuugua
DQ903676                 GGGC-AAUC-UGCAGCCAAGCUCGUACGAGUGCAGUUCACAGACUAUAUGGCAGUGGGUGGCUGCUUAAGACAUAGUCGGUUCGuugua
JQ031957                 GGGA-AACC-UGCAGCCAAGCCGCUACGUGGAAGGAUCACAGACUAAACGACAGUGGGUGGCUGCUUAAAAUAUAGCCGGUAUGuugua
JQ031959                 GGGC-AACC-CGCAGCCAAGUCCUAACGGAUGCAGUUCACAGACUAGACGAUAGUGGGUGGCUGCUUAAGAUAUAGCCGAUAAGuugua
JQ031960                 GGGU-UAUC-UGCAGCUAAGUCCCAUGGGAUGUAGUACACAGACUAAACGGUAGUGGGUGGCUGCUUAAGACAUAGUCGGAUCGuugua
DQ903683                 GAGU-AAUC-UGCGGCCAAUGUCUAACGGACGGCAGUCACAGACUAGAUGGGAGUGGGCGGCUGCUUAAGGAAUAGUCGGUACGuugua
DQ903673                 GGGC-AAUC-CGCAGCCAAAUUCUACCGAAUGCAGUCCCCUGAAUAAACGGUAGUGGGUGGCUGCUUAAGAUAUAGUCGAGGGGuugua
JQ031966                 GGGU-UAUC-UGCAGCCAAGCCCGUCAGGGUGCAGUUCACAGACUAAAUGUUAGUGGGUGUUCGCUUAAGAUAUAGUCGGAUCGuugua
JQ031964                 AGGU-GACC-UGUAGCCAAGUUCCUAGGGAUGCAG-CAACUGACCAAAAGCCAGUGGGUGGUCGCUUAAGAUAUAGUCGGAUCGuugua
JQ031969                 GGGU-GAUC-CGCAGCCAAGCCCGCAAGGGUGCAGUUCACAGACUAAAUGUUAAUGGGUGGCUGCUUAAGAUAUAGCCGGAUCGuugua
JQ031973                 GGGA-UACC-UGCAGCCAAGUCCUGAUGGAUGCAGUUCACAGGCUAUACGGUGGUGGGUGGCUGCUUAAGACUUAGUCGGGCUGuugua
JQ031976                 GGGU-UAUC-UGCAGCCAAGCCCUACUGGGCGCAGUCCACAGACUAAACGUUAGUGGGUGGCUGCUUAAGAUAUAGUCGGCGUGuugua
HQ687204                 GGGU-UACC-UGCAGCCAAGUCCUCGUGGAUGCAGUUCACAGGCUAAACGGUGGUGGGUGGCUGCUUAAGAUAUAGUCGGAUCGuugua
HQ687196                 GGGU-UACC-GGCAGCCAAGCCCGUCAGGGUGCAGUUCACUGGCUAAACGUAGGUGGGUGAUUGCUUAAGAUAUAGUCGAACCGuugua
HQ687197                 GGGU-AACC-UGCAGCCAAGUCCUUGUGGAUGCAGUUCACAGGCUAAACGGUGGUGGGUAGCUGCUUAAGAUAUAGUCGGAUCGuugua
HQ687200                 GGGU-UACC-UGCAGCCAAGUCCGUACGGAUGCAGUUCACAGACUAAACGUCAGUGGGUGGCUGCUUAAGAUAUAGCCGGUAUGuugua
JQ031978                 GGGU-UAUC-UGCAGCUAAGCCCGCCAGGGUGAUGUUCACAGACUAAACGGCAGUGGGUAGCUGCUUAAGAUAUAGUCGAUAUGuugua
JQ031980                 GGAC-AACC-UGCAGCCAAGUCCGUGAGGAUGCAGCUCACAGACUAAACGUCAGUGGGUGGCUGCUUAAGACAUAGCCGGUACGuugua
JQ031979                 GGAC-AACC-UGCAGCCAAGUCCGUGAGGAUGCAGCUCACAGACUAAACGUCAGUGGGUGGCUGCUUAAGAUAUAGCCGGUACGuugua
JQ031985                 GGGU-UAUC-UGCAGCCAAGCCCGCAAGGGUGCAGUUCACAGACUAGAUGUUAGUGGGUAGCUGCUUAAGAUAUAGCCGGAUCGuugua
HQ687194                 UGGC-AACC-UGCAGCCAAGUCCUAACGGAUGCAGUUCACAGGCCAAACGGUGGUGGGUGGCUGCUUAAGAUAUGGUCGGAGAGuugua
HQ687202                 UGGC-AACC-UGCAGCCAAGUCCUAACGGAUGCAGUUCACAGACCAAACGGUGGUGGGUGGCUGCUUAAGACAUGGUCGUCGAGuugua
HQ687195                 GGGC-AACC-UGCAGCCAAGUCCUAACGGAUGCAGUUCACAGACCAAACGGUGGUGGGUGGCUGCUUAAGACAUGGUCGUCGAGuugua
DQ903674                 GGGC-UACC-UGCAGCCAAGCCCGCCAGGGUACAGUUCACAGACUAAAUGGCAGUGGGUGGCUGCUUAAGACAUAGUCGAAGAGuugua
DQ903672                 GGGU-UAUC-CGCAGCCAAGCCCGUUAGGGUGCAGUUCACAGACUAAAUGUUAGUGGGUGUUCGCUUAAGAUAUAGUCGGACAGuugua
JQ031999                 GGGU-UAUC-UGCAGCCAAACCCCACGGGGCCCAGUUCACAGACUAAAUGGUAGUGGGUGGCCGCUUAAGAUGUAGUCGCAGAGuugua
DQ903671                 GGGU-UAUC-UGCAGCCAAACCCCACGGGGCCCAGUUCACAGACUAAAUGGUAGUGGGUGGCCGCUUAAGAUGUAGUCGCAGAGuugua
JQ032000                 GGGU-UAUC-UGCAGCCAAACCCCACGGGGCCCAGUUCACAGACUAAAUGGUAGUGGGUGAUCGCUUAAGAUAUAGUCGCAGAGuugua
JX481296                 GGGC-UAUC-CGCAGCCGUGCCCCCGGGGGCGCAGUUCACAGACUAAAUGUUGGUGGGUGGUCGCCUAAGAUAUAGCCGAUGAGuugua
KM494993                 GGGU-AACC-CGCAGCCAAGCCCGUACGGGUGCAGUUCACAGACUAAAUGUCGGUGGGUGGCUGCUUAAGAUAUAGCCGGGCUGuugua
KM494994                 GGGU-GACC-CGCAGCCAAGCCCGUACGGGUGCAGUCCACAGACUAAAUGCGGGUGGGUGGCUGCUUAAGAUAUAGCCGGGCUGuugua
KM494995                 GGGU-GACC-CGCAGCCAAGCCCGUACGGGUGCAGUCCACAGACUAAAUGUCGGUGGGUGGCUGCUUAAGAUAUAGCCGGGCUGuugua
KM494996                 GGGU-AACC-CGCAGCCAAGCCCGUACGGGUGCAGUUCACAGACUAAAUGUCGGUGGGUGGCUGCUUAAGAUAUAGCCGGGCUGuugua
KM494997                 GGGU-AACC-CGCAGCCAAGCUCGUACGAGUGCAGUUCACAGACUAAAUGUUGGUGGGUGAUCGCUUAAGAUAUAGUCGGCGUGuugua
KM494998                 GGGU-AACC-CGCAGCCAAGCUCGUACGAGUGCAGUUCACAGACUAAAUGUUGGUGGGUGAUCGCUUAAGAUAUAGUCGGCGUGuugua
KM494999                 GGGU-AACC-CGCAGCCAAGCUCGUACGAGUGCAGUUCACAGACUAAAUGUUGGUGGGUGAUCGCUUAAGAUAUAGUCGGCGUGuugua
KM495003                 GGGU-AACC-CGCAGCCAAGCUCGUACGAGUGCAGUUCACAGACUAAAUGUUGGUGGGUGAUCGCUUAAGAUAUAGUCGGCGUGuugua
KM495005                 GGGU-AACC-CGCAGCCAAGCUCGUACGAGUGCAGUUCACAGACUAAAUGUUGGUGGGUGAUCGCUUAAGAUAUAGUCGGCGUGuugua
KM495006                 GGGU-AACC-CGCAGCCAAGCUCGUACGAGUGCAGUUCACAGACUAAAUGUUGGUGGGUGAUCGCUUAAGAUAUAGUCGGCGUGuugua
KM495009                 GGGU-AACC-CGCAGCCAAGCUCGUACGAGUGCAGUUCACAGACUAAAUGUUGGUGGGUGAUCGCUUAAGAUAUAGUCGGCGUGuugua
KM495010                 GGGU-AACC-CGCAGCCAAGCUCGUACGAGUGCAGUUCACAGACUAAAUGUUGGUGGGUGAUCGCUUAAGAUAUAGUCGGCGUGuugua
KM495018                 GGGU-AACC-CGCAGCCAAGCUCGUACGAGUGCAGUUCACAGACUAAAUGUUGGUGGGUGAUCGCUUAAGAUAUAGUCGGCGUGuugua
KM495019                 GGGU-AACC-CGCAGCCAAGCUCGUACGAGUGCAGUUCACAGACUAAAUGUUGGUGGGUGAUCGCUUAAGAUAUAGUCGGCGUGuugua
KM495020                 GGGU-AACC-CGCAGCCAAGCUCGUACGAGUGCAGUUCACAGACUAAAUGUUGGUGGGUGAUCGCUUAAGAUAUAGUCGGCGUGuugua
KM495021                 GGGU-AACC-CGCAGCCAAGCUCGUACGAGUGCAGUUCACAGACUAAAUGUUGGUGGGUGAUCGCUUAAGAUAUAGUCGGCGUGuugua
KM495022                 GGGU-AACC-CGCAGCCAAGCUCGUACGAGUGCAGUUCACAGACUAAAUGUUGGUGGGUGAUCGCUUAAGAUAUAGUCGGCGUGuugua
KM495023                 GGGU-AACC-CGCAGCCAAGCUCGUACGAGUGCAGUUCACAGACUAAAUGUUGGUGGGUGAUCGCUUAAGAUAUAGUCGGCGUGuugua
KM495024                 GGGU-AACC-CGCAGCCAAGCUCGUACGAGUGCAGUUCACAGACUAAAUGUUGGUGGGUGAUCGCUUAAGAUAUAGUCGGCGUGuugua
KM495026                 GGGU-AACC-CGCAGCCAAGCUCGUACGAGUGCAGUUCACAGACUAAAUGUUGGUGGGUGAUCGCUUAAGAUAUAGUCGGCGUGuugua
KM495027                 GGGU-AACC-CGCAGCCAAGCUCGUACGAGUGCAGUUCACAGACUAAAUGUUGGUGGGUGAUCGCUUAAGAUAUAGUCGGCGUGuugua
KM495028                 GGGU-AACC-CGCAGCCAAGCUCGUACGAGUGCAGUUCACAGACUAAAUGUUGGUGGGUGAUCGCUUAAGAUAUAGUCGGCGUGuugua
KM495029                 GGGU-AACC-CGCAGCCAAGCUCGUACGAGUGCAGUUCACAGACUAAAUGUUGGUGGGUGAUCGCUUAAGAUAUAGUCGGCGUGuugua
KM495030                 GGGU-AACC-CGCAGCCAAGCUCGUACGAGUGCAGUUCACAGACUAAAUGUUGGUGGGUGAUCGCUUAAGAUAUAGUCGGCGUGuugua
JX481284                 GGGU-AACC-CGCAGCCAAGCCCGUACGGGUGCAGUUCACAGACUAAAUGGUGGUGGGUAUUUGCUUAAGAUAUAGCCGGCGUGuugua
V01416                   UCCU-AACCACGCAGCCAAGUCCUAAUGGAUGCAGUUCACAGACUAAAUGUCGGUCGGGGAUCUCAUAAGAUAUAGUCGGAUCGuaagg
MZ313554*                GGGU-AAUC-CGCAGCCAAGUCCGUACGGAUGCAGUUCACAGACUAAAAGGUGGUGGGUGAUUGCUUAAGAUAUAGUCGAAAGGcagau
```

Figure S2: Sequence alignment of core structure nucleotides of myxomycete S1389 group I intron. Dashes correspond to deleted positions. Secondary structure paired segments (P1-P8) are shown above the alignment. Intron sequences are indicated by GenBank accession numbers. V01416 and MZ313554* correspond to the out-group *Tetrahymena* intron Tth.L1925 and *Mucilago* intron Mcr.S788, respectively.

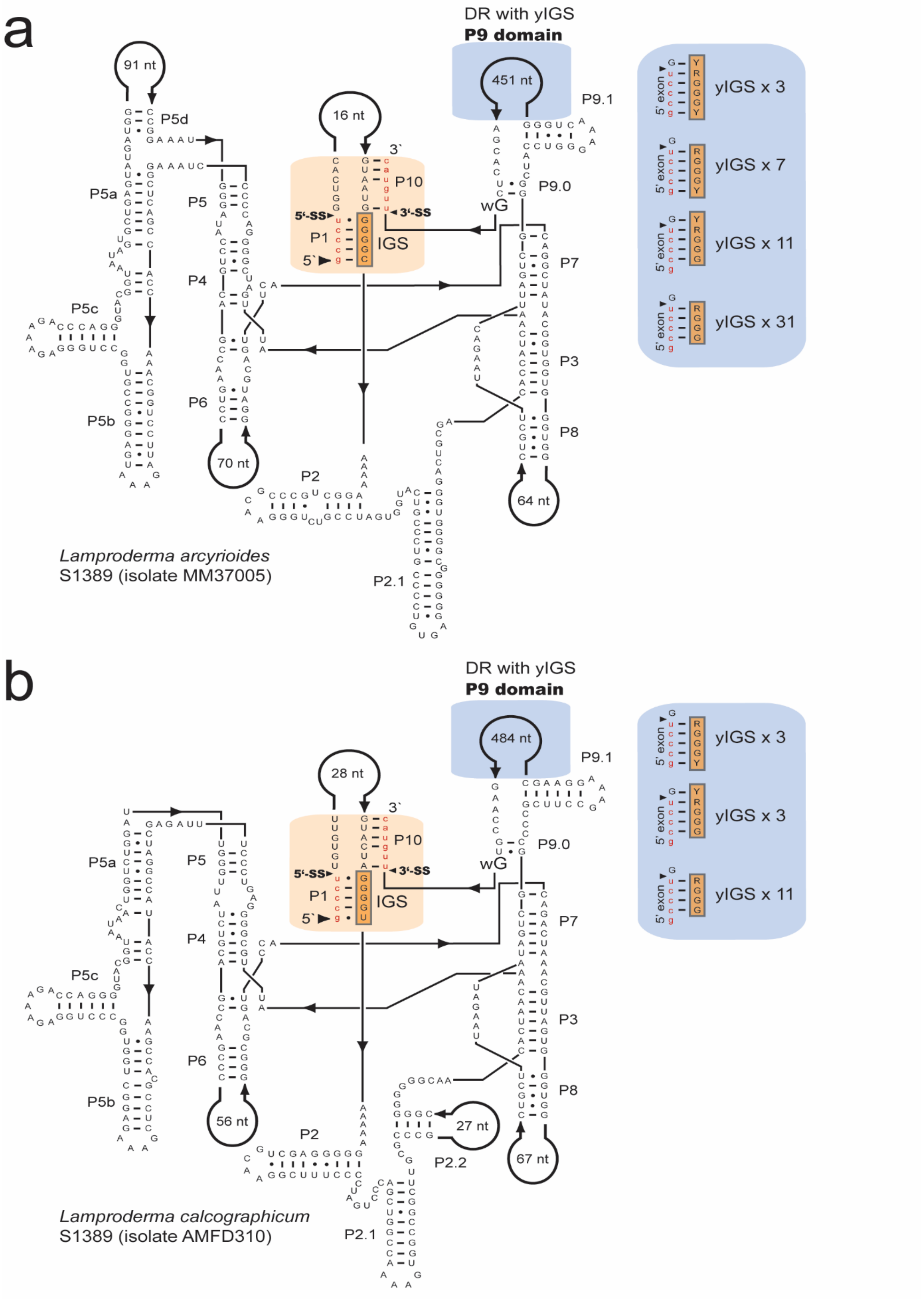

Figure S3

Figure S3: Secondary structure diagrams of (**a**) *Lamproderma arcyriodies* and (**b**) *Lamproderma arcyriodies* S1389 group I intron. P1-P10, paired RNA segments; 5' SS and 3'SS, exon-intron splice sites; IGS, internal guide sequence. The introns contain large extensions in P9 that contains direct repeat motifs (blue box) with ΨIGS sequences motifs.

a

His-Cys Box homing endonuclease (KM494996)

MPPKESKAEKKMREHRNETKRRSRAKIKRDQDEKRRKLGLPPPERGATSANRAARLGLLLCATDAGLLEMRGQSQGEVTHGTCVYVPRLR
MHKRMVDKARLEVYREVRETVELMGEDEKVQAHHVQMILDDRERVGRWYDHLKARAAALANGEAGNLPDVTGSHTCRKELFMQSLSGGER
ARYRDYTCAAPFHCNPDYHSHNLAQKKCPSQHRFRVAGALYRADCTCRAPCVLSDNQVIDLKPE (244 amino acids)

His-Cys Box homing endonuclease (KM495030)

MPRHTTEETTEEALERKAKNKRASRATLKLQREKGSEKPAKKARGATAENKANRLRLVLCQTDLKLLALRAAAVKTGVEFGPCLYAPRVR
LSKDKTVWDIERMKRFGVTRETVRALGEDEKVQAHHVQMVLDDRVRVEEWYDRLVSRAAAISKGDAEVPPDYTGSHTCRKEAFRQSLDPK
EQAKYRDHTCTAQFHCVPVLHSVNLAQKDCPSKHRFLLNGEIYRAECQCSVRCVPFQTNTTLSLVKE (247 amino acids)

b

Homing endonuclease gene (KM494996)

AUGCCGCCUAAGGAAAGCAAAGCAGAGAAAAAAAUGAGGGAGCAUAGGAAUGAGACUAAACGCAGAAGCCGGGCAAAGAUCAAGCGAGACCAAGAUGAGAAAAGGCGGAAGCUAGGGUUG
CCCCCCCCUGAGAGGGGGGCGACCUCGGCUAACAGGGCGGCUAGGUUGGGCCUGCUUCUGUGCGCGACUGAUGCUGGCCUCUUAGAGAUGCGGGGCCAGAGCCAAGGGGAAGUGACGCAC
GGCACAUGCGUAUAUGUUCCUCGCCUUAGAAUGCACAAGGUAGGCCGUGUAGGCCCGCCCACCCCUGCUAAUGCAUCAAACAGCGCAUGGUUGAUAAGGCUCGCUUGGAAGUGUACCGGG
AGGUACGGGAAACCGUUGAGCUCAUGGGCGAAGAUGAGAAGGUACAGGCGCACCACGUGCAAAUGAUCCUAGAUGAUCGUGAGCGUGUGGGGCGCUGGUAUGACCAUCUAAAAGCCCGGG
CUGCUGCCCUUGCUAAUGGCGAGGCAGGCAGGAAACCUCCCGGAUGUCACUGGGUCGCAUACCUGCCGCAAGGAGCUGUUUAUGCAGUCUCUAAGCGGGGGGGAGAGGGCGCGAUACAGG
GACUAUACCUGUGCUGCGCCUUUUCAUUGCAAUCCCGACUAUCACUCCCAUAACCUAGCCCAGAAGAAGUGCCCAUCUCAACAUAGGUUUCGAGUGGCUGGCGCCUUGUAUAGGGCCGAC
UGCACGUGCAGAGCGCCCUGCGUAUUAAGCGAUAAUCAAGUAAUAGACCUCAAACCCGAAUAAAAUCU

Homing endonuclease gene (KM495030)

AUGCCAAGACACACAACAGAAGAAACCACAGAAGAAGCCCUGGAACGUAAGGCUAAGAACAAGCGUGCAUCAAGGGCAAGGGCAACGUUGAAGCUGCAGAGGGAGAAAGGCUCUGAAAAG
CCUGCCAAGAAAGCAAGGGGGGCUACGGCCGAGAACAAGGCCAAUAGGCUGCGUCUCGUCCUAUGCCAGACCGACCUCAAGCUAUUGGCCCUCCGUGCUGCAGCUGUCAAGACUGGGGUG
GAGUUUGGUCCGUGCCUUUAUGCGCCUCGUGUGAGGCUAUCAAAGGUAUGUAUGGGGUUGGGACUUGUUGGGGCUUUUCUGACUGAUUCCUCCUGUAGGAUAAGACUGUCUGGGAUAUCG
AGCGCAUGAAAAGGUUUGGGGUGACACGGGAAACCGUUCGUGCCCUGGGAGAAGACGAGAAGGUACAGGCUCACCAUGUGCAGAUGGUCUUAGAUGAUCGUGUGCGCGUGGAGGAGUGGU
AUGACCGUUUAGUAUCCCGGGCCGCUGCAAUCAGCAAAGGCGAUGCAGAAGUCCCCCCCGACUACACGGGAUCCCACACAUGCCGGAAAGAGGCUUUUAGGCAGAGCCUCGAUCCGAAGG
AGCAGGCCAAGUACAGGGAUCAUACCUGUACGGCCCAGUUCCACUGUGUGCCCGUUUUGCACUCGGUAAACCUGGCACAGAAGGAUUGCCCCAGUAAACACAGGUUCCUUCUUAAUGGGG
AAAUAUACCGGGCGGAAUGCCAGUGUUCUGUCCGGUGUGUGCCCUUCCAGACCAAUACGACUUUAUCCCUUGUAAAAGAAUAAAAUGC

c

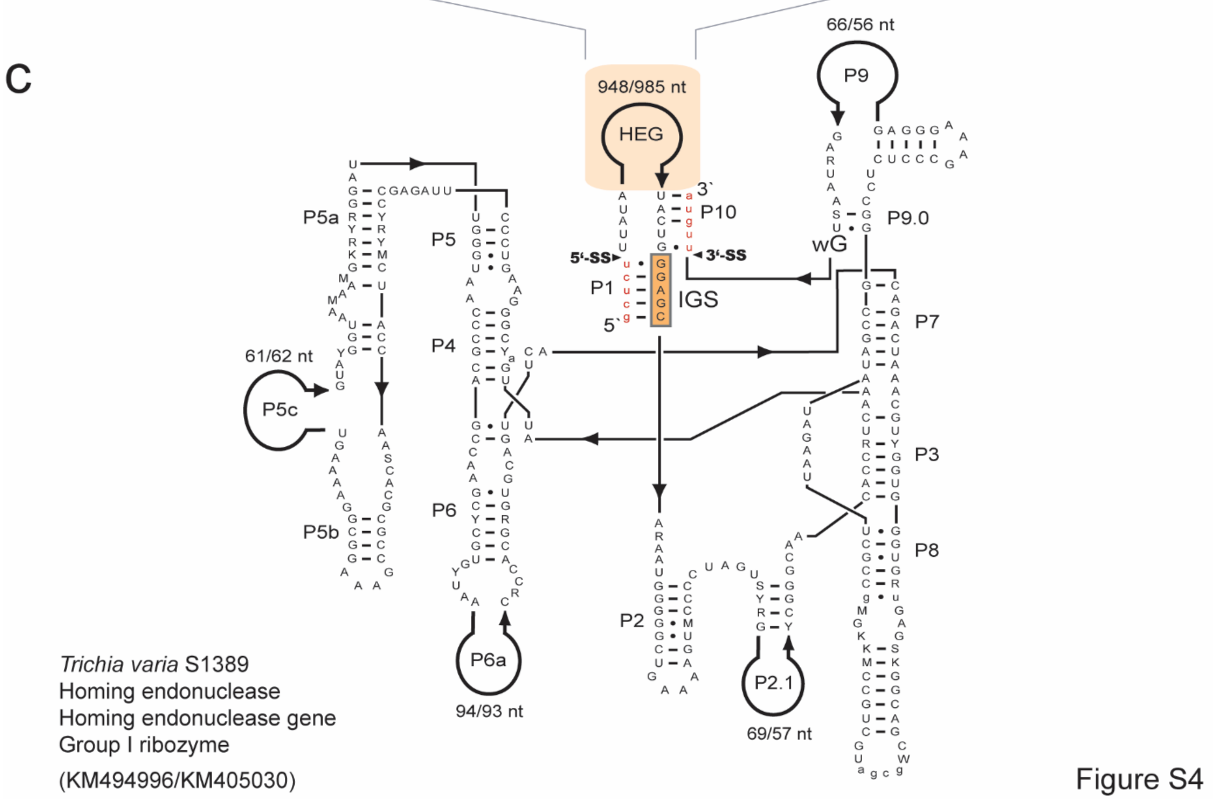

Figure S4: Sequence features in *Trichia varia* S1389 group I introns. (**a**) Amino acid sequence of *T. varia* homing endonucleases, including His-Cys box motifs (green letters). (**b**) Nucleotide sequence of *T. varia* homing endonuclease genes, including spliceosomal introns (red letters). Start codon (AUG) and stop codon (UAA) are indicated by green letters. Polyadenylation signal (AAUAAA) is indicated as bold underlined letters at the 3' end of sequence. (**c**) Secondary structure

diagram of *T. varia* group I introns (KM494996 and KM495030). Homing endonuclease gene (HEG) insertions are located in segment P1. P1-P10, paired RNA segments; 5' SS and 3'SS, exon-intron splice sites; IGS, internal guide sequence; ωG, last nucleotide residue of intron; red lowercase letters, exon sequences; black lowercase letters, nucleotide positions only present in one of the introns. R represents A or G (purine); Y represents C or U (pyrimidine); K represents G or U; M represents A or C; S represents C or G; W represents A or U.

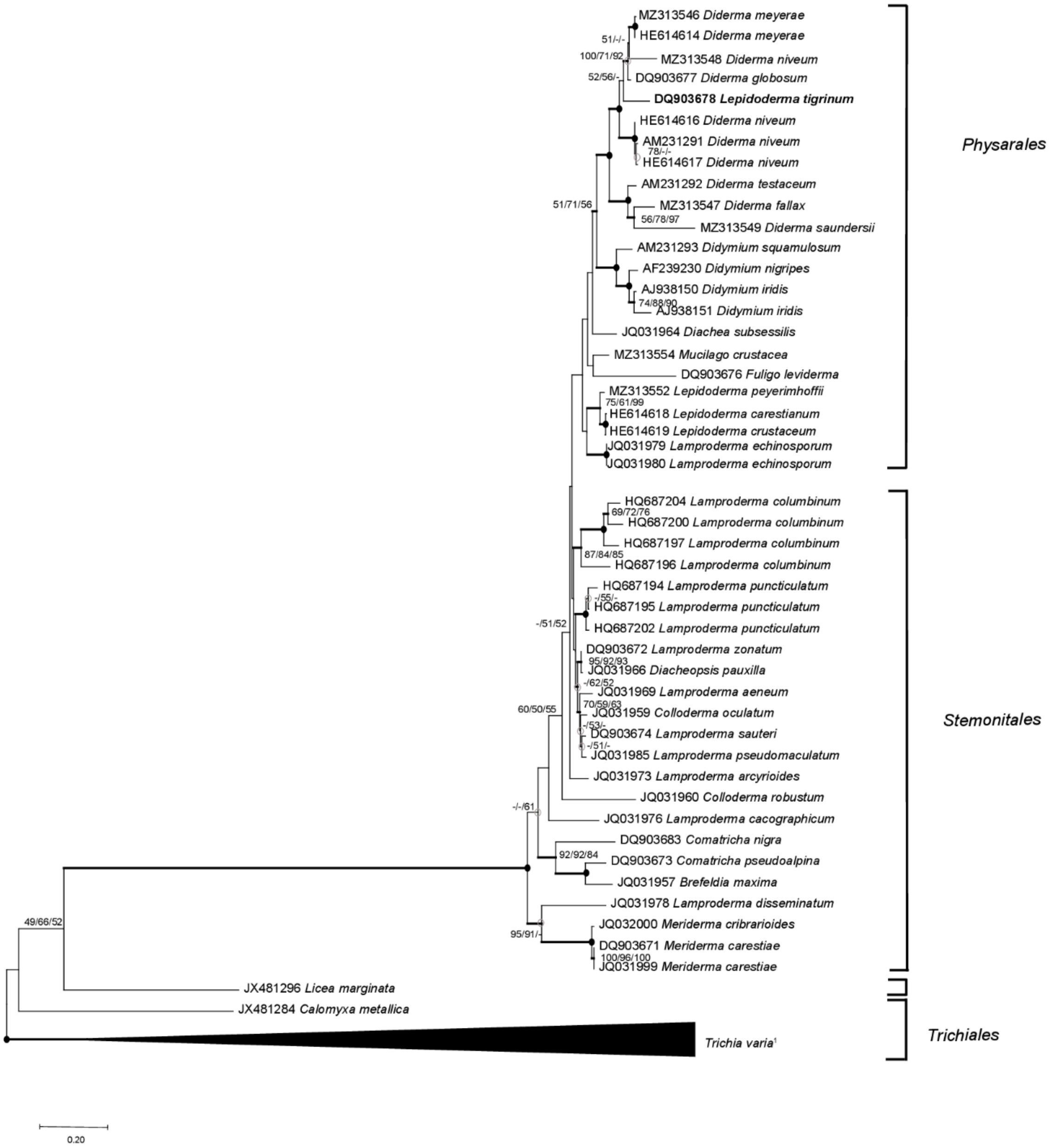

Figure S5: Molecular phylogeny of myxomycete taxa based SSU rDNA sequences. The SSU topology is obtained by neighbor-joining (NJ) analysis of 72 taxa and 1,575 nt aligned positions (SSU dataset 1; Table S1). The tree is rooted with the *T. varia* SSU rDNA sequence. The NJ, maximum likelihood (ML), and maximum parsimony (MP) bootstrap replicates (≥50%) are given at each node. Bayesian posterior (BI) probabilities (≥0.95) are shown in bold branches. Black dots at branch points; maximum support in NJ, ML, and MP (≥97%). The scale bar indicates the fraction of substitutions per site.

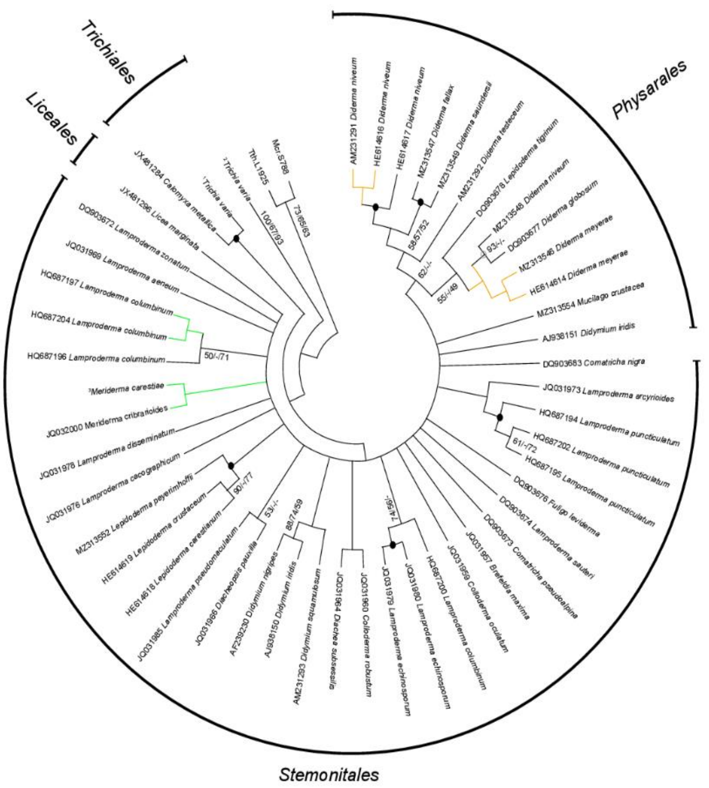

Figure S6: Molecular phylogeny of myxomycete S1389 group I introns. The intron topology is obtained by neighbor-joining (NJ) analysis of 74 taxa (including group introns from *Tetrahymena* (Tth.1925) and *Mucilago* (Mcr.S788), and 166 nt aligned positions (intron dataset 2; Table S1). The NJ-, maximum likelihood (ML), and maximum parsimony (MP) bootstrap replicates (≥ 50%) are given for each node. Bayesian posterior (BI) probabilities (≥0.95) are shown in bold branches. Black dots at branch site indicate maximum support in NJ, ML, and MP (≥98%). Green line indicates maximum support in NJ, ML and MP (≥90%). Orange line indicates maximum support in NJ, ML, and MP (≥70%). Gray circles indicate alternative topologies in NJ, ML, and MP. [1] *Tricia varia* clade is composed of four *T. varia* isolates (KM494993-6). [2] *Tricia varia* clade is composed of twenty *T. varia* isolates (KM494997-9, KM495003, KM495005, KM495006, KM495009, KM495010, KM495018-30). [3] *Meriderma carestiae* clade is composed of two *M. carestiae* isolates (JQ031999 and DQ903671). The scale bar indicates the fraction of substitutions per site.
